# A compartment model of VEGF distribution in blood, healthy and diseased tissues

**DOI:** 10.1186/1752-0509-2-77

**Published:** 2008-08-19

**Authors:** Marianne O Stefanini, Florence TH Wu, Feilim Mac Gabhann, Aleksander S Popel

**Affiliations:** 1 Department of Biomedical Engineering, School of Medicine, Johns Hopkins University, Baltimore, Maryland 21205, USA; 2 Department of Biomedical Engineering, University of Virginia, Charlottesville, VA 22908, USA; 3 Robert M. Berne Cardiovascular Research Center, University of Virginia, Charlottesville, VA 22908, USA

## Abstract

**Background:**

Angiogenesis is a process by which new capillaries are formed from pre-existing blood vessels in physiological (e.g., exercise, wound healing) or pathological (e.g., ischemic limb as in peripheral arterial disease, cancer) contexts. This neovascular mechanism is mediated by the vascular endothelial growth factor (VEGF) family of cytokines. Although VEGF is often targeted in anti-angiogenic therapies, there is little knowledge about how its concentration may vary between tissues and the vascular system. A compartment model is constructed to study the VEGF distribution in the tissue (including matrix-bound, cell surface receptor-bound and free VEGF isoforms) and in the blood. We analyze the sensitivity of this distribution to the secretion rate, clearance rate and vascular permeability of VEGF.

**Results:**

We find that, in a physiological context, VEGF concentration varies approximately linearly with the VEGF secretion rate. VEGF concentration in blood but not in tissue is dependent on the vascular permeability of healthy tissue. Model simulations suggest that relative VEGF increases are similar in blood and tissue during exercise and return to baseline within several hours. In a pathological context (tumor), we find that blood VEGF concentration is relatively insensitive to increased vascular permeability in tumors, to the secretion rate of VEGF by tumors and to the clearance. However, it is sensitive to the vascular permeability in the healthy tissue. Finally, the VEGF distribution profile in healthy tissue reveals that about half of the VEGF is complexed with the receptor tyrosine kinase VEGFR2 and the co-receptor Neuropilin-1. In diseased tissues, this binding can be reduced to 15% while VEGF bound to the extracellular matrix and basement membranes increases.

**Conclusion:**

The results are of importance for physiological conditions (e.g., exercise) and pathological conditions (e.g., peripheral arterial disease, coronary artery disease, cancer). This mathematical model can serve as a tool for understanding the VEGF distribution in physiological and pathological contexts as well as a foundation to investigate pro- or anti-angiogenic strategies.

## Background

Angiogenesis is the process by which new blood vessels grow from pre-existing vascular networks. This is a dynamic physiological mechanism (e.g., during wound healing, exercise training) but has been shown to be involved in pathological conditions such as age-related macular degeneration, rheumatoid arthritis, diabetic retinopathy, peripheral arterial disease, and cancer [1]. Vascular endothelial growth factor (VEGF) is a potent cytokine involved in angiogenesis [2]. This growth factor plays a role in transcapillary permeability and stimulates cell differentiation, proliferation, migration and survival. The human VEGF gene family is composed of five members. VEGF-A (also commonly referred as VEGF) and VEGF-B are known to be involved in angiogenesis. VEGF-A and three members (VEGF-C, VEGF-D and placental growth factor PlGF) have been shown to be implicated in both mechanisms. The VEGF-A family contains the different splice isoforms VEGF_121_, VEGF_121_b, VEGF_145_, VEGF_145_b, VEGF_148_, VEGF_162_, VEGF_165_, VEGF_165_b, VEGF_183_, VEGF_189_, VEGF_206_, VEGF_232 _[3]. The major splice variants are VEGF_121_, VEGF_165_, VEGF_189 _and VEGF_206_. Because the role of VEGF_189 _and VEGF_206 _in vivo is not well understood and their expression is low compared to VEGF_121 _and VEGF_165 _[3], we consider the two main isoforms VEGF_121 _and VEGF_165 _in our model. These two ligands bind two receptor tyrosine kinases VEGFR1 (Flt-1, fms-like tyrosine kinase-1) and VEGFR2 (Flk-1/KDR, fetal liver kinase-1/Kinase Domain-containing Receptor). The VEGF_165 _isoform binds to a non-signaling co-receptor called Neuropilin-1 (NRP1) and to heparan sulfate proteoglycans which are constituents of the extracellular matrix (ECM) and the cellular basement membranes. NRP1 also independently couples with VEGFR1, to which VEGF_121 _can bind to form a complex of all three. Another complex can be formed when VEGF_165 _bound to NRP1 binds in turn to VEGFR2 (or vice versa).

VEGF interactions with these receptors in the context of angiogenesis have been extensively studied using computational models. For example, it was predicted that blockade of NRP1-VEGFR coupling would be a good strategy to decrease VEGF-VEGFR2 signaling [4]. Other simulations showed that VEGFR heterodimers comprise 10–50% of the active, signaling VEGF receptor complexes, and that heterodimers form at the expense of homodimers of VEGFR1 when VEGFR2 populations are larger [5]. However, in the absence of in vivo data, we do not include heterodimers in the model. Insights into the distribution of VEGF in tissues in vivo have been made; for example, it was predicted that the majority of the VEGF in muscle is bound to the cell surface receptors or to the extracellular matrix and that NRP1 enhances the binding of VEGF_165 _to VEGFR2 [6]. The models have also been used to design and test therapeutic approaches; for example, in peripheral arterial disease, exercise training results in increased VEGF secretion in hypoxic tissue and augmented VEGF receptor expression, and multi-scale computational models revealed that this leads to an increase in both VEGF concentration and VEGF gradients, a potentially effective therapeutic approach [7]. Anatomically detailed tissue models have predicted heterogeneity in the activation of the endothelial VEGF receptors and how this affects VEGF gradients [8]. Similarly, in hypoxic tissues, the high spatial heterogeneity of muscle fiber VEGF secretion leads to significant gradients of VEGF concentration and VEGF receptor activation in both resting and exercising muscle [9]. Three-dimensional VEGF distribution was also predicted in ischemic muscle in a model of peripheral arterial disease [10].

A meta-analysis of VEGF levels in healthy subjects and cancer patients revealed that plasma VEGF levels are several-fold higher in cancer [11]. This conclusion should be taken cautiously since VEGF levels vary with the tumor type, size and location; also, the ranges of VEGF in control subjects and cancer patients in some studies overlap. Plasma VEGF is also elevated during exercise [12,13] and in peripheral arterial disease [14]. To investigate the distribution of VEGF in human subjects under physiological and pathological conditions, we formulate a biophysically-accurate compartment model to describe the entire human body. Blood is represented by one compartment that communicates with two others representing healthy and diseased tissues. A sensitivity analysis is performed to investigate the role of parameters including secretion rate, clearance rate and vascular permeability of VEGF. The formulation is general and the model can be applied to both healthy human subjects and to subjects with a diseased tissue, e.g., ischemic limb or tumor. As an illustration, we consider a compartment representing a tumor to investigate the possible causes of the several-fold increase observed in plasma VEGF levels in cancer patients [11]. The formulated model provides a foundation for studying various diseases where information about VEGF distribution in the body is important. It will also serve as a basis for simulating pro- and anti-angiogenic VEGF-related therapeutic procedures.

## Methods

### Geometry

**Figure 1:**
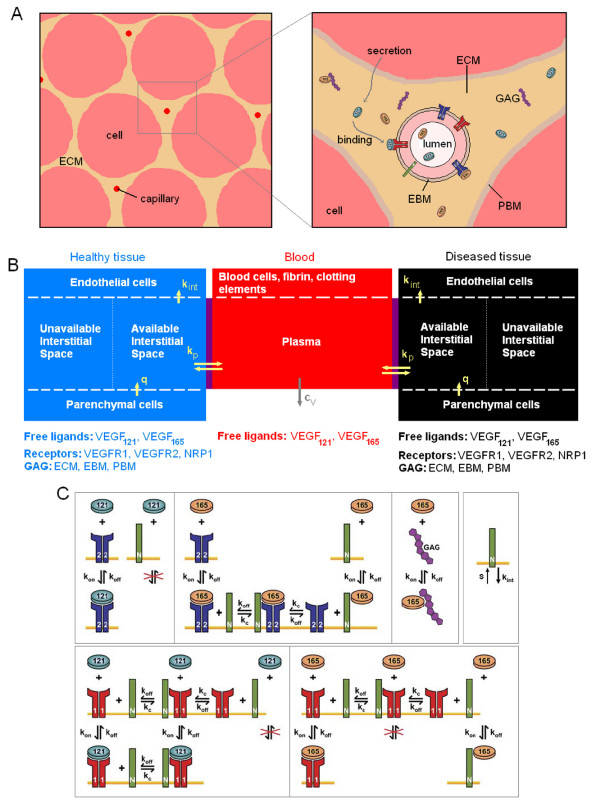
**Compartment model of VEGF transport in blood and tissues**. **A**, Schematic of a tissue cross section. VEGF_165 _can bind to glycosaminoglycan chains (GAG) and be sequestered in the extracellular matrix whereas VEGF_121 _cannot. The isoforms have different cell surface receptor binding profiles. **B**, Compartment model set-up. Three compartments are used in our simulations: blood, healthy and diseased tissues. The diseased tissue compartment is not used in all simulations. VEGF is secreted by parenchymal cells in the healthy and diseased compartments (*q*). VEGF transport between the blood and the tissue compartments is via transcapillary permeability (*k*_*p*_). VEGF receptors are expressed on the abluminal side of the endothelial cells and VEGF binding to these receptors can lead to internalization (*k*_*int*_). Plasma clearance for VEGF is present in the blood compartment (*c*_*V*_). **C**, Interactions between VEGF, cell surface receptors, extracellular matrix and basement membranes. VEGF_121 _binds to VEGFR2 but does not bind to NRP1. VEGF_165 _interacts with VEGFR2 or NRP1. Once bound, it can form a ternary complex VEGFR2- VEGF_165_- NRP1. VEGF_165 _can be sequestered by the ECM, PBM or EBM by binding to GAG chains. VEGF_121 _binds to VEGFR1. This receptor couples with NRP1 to form VEGFR1-NRP1 complex or the ternary complex VEGF_121_-VEGFR1-NRP1 if previously occupied by VEGF_121_. VEGF_165 _also binds to VEGFR1 but not to the VEGFR1-NRP1 complex. The receptors (VEGFR1, VEGFR2 and NRP1) are inserted or internalized at the cell surface.

As a first approximation, a tissue can be represented as a collection of capillaries (and small arterioles and venules), surrounded by parenchymal cells. For example, skeletal muscle is constituted of long fibers whose cross sections are approximately constant. A schematic of this configuration is shown in Figure 1A. Note that the stromal cells are not considered explicitly in the model, but rather lumped with parenchymal cells. Between the parenchymal cells and the capillaries lies the interstitial space composed of the extracellular matrix (ECM), parenchymal basement membranes (PBM) and endothelial basement membranes (EBM). In this study, these anatomical structures will be represented in a spatially-averaged manner: each structure will be represented by a distinct volume with specific VEGF binding properties, but VEGF gradients within the volume will not be considered.

### Computational model

We constructed a compartment model of VEGF transport and interactions with cell receptors representing the whole human body that is an extension of previous one-compartment models for breast tumor tissue [4] and skeletal muscle [6]. This design can also be used to describe any animal if the parameters to characterize the tissues and blood are available. In physiological cases, we consider two compartments: tissue and blood. VEGF transport between the compartments is mediated by transcapillary permeability. In pathological cases, a third compartment represents the diseased tissue, e.g., a tumor in cancer or ischemic tissue in peripheral arterial disease (Figure 1B). Again vascular permeability allows free VEGF to move between the blood and the diseased tissue. Because the interacting surface between the healthy and the diseased tissues is, in most cases, much smaller than the trans-endothelial exchange surface between each tissue and blood, we do not include VEGF transport between the normal and diseased tissue directly. This additional parameter could be included in further studies.

Leaving aside the vascular system, the tissues of the body can be divided into two main groups: skeletal muscle (35%) [15] and the rest of the body (e.g., brain, heart, liver, kidney, bones, fat). As a first approximation, we assume that the rest of the body has the same characteristics as skeletal muscle. This assumption can be relaxed by formulating progressively more detailed compartment models containing different organs and tissues; however, the parameters describing VEGF-binding properties of different tissues are currently unavailable. Therefore, the healthy tissue compartment in the present model has the mass of the whole body (minus the blood) and the density of the skeletal muscle.

The interstitial space between the parenchymal cells and the vascular endothelial cells can be divided into three regions: the extracellular matrix (ECM), the basement membrane surrounding the parenchymal cells (PBM) and the basement membrane surrounding the endothelial cells (EBM). The two isoforms of the VEGF-A family considered in the model are the freely diffusible heparin-binding VEGF_165 _(VEGF_164 _in rodents) and non-heparin-binding VEGF_121 _(VEGF_120 _in rodents). Because VEGF_165 _binds to the heparan sulfate proteoglycans (HSPGs) that constitute a major component of the extracellular matrix and basement membranes, this isoform can be sequestered by the ECM and by cellular basement membranes. We assume that the distribution of unbound (free) VEGF in the interstitial space is uniform within each compartment and spatial variability due to gradient formation is neglected [4,6,16].

The interactions between VEGF_121 _and VEGF_165 _and their receptors (VEGFR1, VEGFR2 and NRP1) are illustrated in Figure 1C and will be further described in the equation section. To summarize, VEGF_121 _and VEGF_165 _bind to both VEGFR1 and VEGFR2 but only VEGF_165 _is believed to bind to the non-enzymatic co-receptor NRP1. The receptors are inserted or internalized as illustrated in Figure 1C. We assume that the receptors are only present on the abluminal side of the blood vessels and therefore are only exposed to interstitial VEGF levels of the tissue compartments, though this assumption can be easily relaxed in the future. Although there is some qualitative evidence that receptors are present on the luminal side [17], to our knowledge, there is currently no quantitative data on the levels of receptor expression on the luminal vs. abluminal side of the endothelium.

VEGF molecules are secreted by the parenchymal cells present in the tissue. Depletion of VEGF molecules occurs if the cell surface receptor (VEGFR1, VEGFR2 or NRP1) that VEGF is bound to is internalized into the cellular membrane. The free molecules diffuse within the available interstitial fluid although here this diffusion is assumed to be fast compared to the biochemical reactions, the compartment well-mixed and the distribution of molecules uniform; the justification for this assumption was presented in [7] by estimating that the Damkohler number (ratio of diffusion time to reaction time) is significantly less than one. Free VEGF is transported by transcapillary permeability between the blood and tissue compartments and cleared from the blood (e.g., by the kidneys and liver).

### Equations

The changes in species concentration over time are governed by mass balance relationships and represented by a system of coupled nonlinear ordinary differential equations; the details of the derivation are given in our previous publications [4,6,16]. Although the kinetics and properties may be different, the healthy and diseased tissues are governed by the same equations and constituted by the same molecular species. Therefore, unless specified otherwise, the following equations govern both healthy and diseased tissues. The biological and physical properties of the tissue, VEGF secretion rates and vascular permeability may vary between the normal and diseased tissues; the values of the parameters will be specified in a later section.

Each tissue compartment can be divided into subcompartments where distinct reactions take place: cell surfaces and interstitial space, the latter further subdivided into available interstitial fluid, ECM, PBM and EBM. Interstitial species include free VEGF, VEGF binding sites in the matrix and the complexes they form. Surface species consist of free receptors or VEGF-ligated receptors on the vascular endothelial abluminal surface. Unless specified otherwise, the concentrations of the interstitial and blood species are expressed per unit volume of the corresponding subcompartment (e.g., available fluid volume of the interstitial space, ECM, PBM or EBM) while the concentrations of the surface species are expressed per unit surface area of the corresponding cell (though it is possible to interconvert the units).

#### Interstitial space

We use the following notation: [*M*] is the concentration of VEGF binding sites of the ECM, PBM or EBM (the location is denoted by the corresponding subscript, e.g., [*M*_*ECM*_]); [*V*] represents the concentration of any unbound VEGF isoform (unless specified by a subscript, e.g., [*V*_*121*_]) in the available interstitial fluid; *k*_*on *_and *k*_*off *_the kinetic rates for binding and unbinding respectively. In the interstitium of the normal and diseased tissues, the following reactions take place

$$V_{165}+M_{ECM}\overset{k_{on,V165,MECM}}{\underset{k_{off,V165,MECM}}{⇌}}V_{165}M_{ECM}$$

$$V_{165}+M_{EBM}\overset{k_{on,V165,MEBM}}{\underset{k_{off,V165,MEBM}}{⇌}}V_{165}M_{EBM}$$

$$V_{165}+M_{PBM}\overset{k_{on,V165,MPBM}}{\underset{k_{off,V165,MPBM}}{⇌}}V_{165}M_{PBM}$$

and are governed by the equations

(1) $$\frac{d[M_{EBM}]}{dt}=-k_{on,V165,MEBM}[V_{165}][M_{EBM}]+k_{off,V165MEBM}[V_{165}M_{EBM}]$$

(2) $$\frac{d[M_{ECM}]}{dt}=-k_{on,V165,MECM}[V_{165}][M_{ECM}]+k_{off,V165MECM}[V_{165}M_{ECM}]$$

(3) $$\frac{d[M_{PBM}]}{dt}=-k_{on,V165,MPBM}[V_{165}][M_{PBM}]+k_{off,V165MPBM}[V_{165}M_{PBM}]$$

(4) $$\frac{d[V_{165}M_{EBM}]}{dt}=k_{on,V165,MEBM}[V_{165}][M_{EBM}]-k_{off,V165MEBM}[V_{165}M_{EBM}]$$

(5) $$\frac{d[V_{165}M_{ECM}]}{dt}=k_{on,V165,MECM}[V_{165}][M_{ECM}]-k_{off,V165MECM}[V_{165}M_{ECM}]$$

(6) $$\frac{d[V_{165}M_{PBM}]}{dt}=k_{on,V165,MPBM}[V_{165}][M_{PBM}]-k_{off,V165MPBM}[V_{165}M_{PBM}]$$

#### Cell surface

[*R*], [*N*] and [*RN*] represent the densities of the unoccupied receptor tyrosine kinases (VEGFR1, VEGFR2), the unoccupied co-receptor (NRP1), and their coupled complexes (VEGFR1 coupled with NRP1), respectively; [*VR*] and [*VN*] are the concentrations of VEGF bound to the VEGF receptors and NRP1, respectively; [*RVN*] (or [*VRN*]) is the concentration of VEGF-bound in the form of the ternary complex (VEGFR2-VEGF_165_-NRP1 or VEGF_121_-VEGFR1-NRP1). *s*_*R *_represents the rate at which the receptors are inserted into the cell membrane and *k*_*int *_the internalization rate of the receptors.

The VEGF_165 _isoform interacts with the receptors through the following reactions

$$V_{165}+R_{1}\overset{k_{on,V165,R1}}{\underset{k_{off,V165R1}}{⇌}}V_{165}R_{1}$$

$$V_{165}+R_{2}\overset{k_{on,V165,R2}}{\underset{k_{off,V165R2}}{⇌}}V_{165}R_{2}$$

$$V_{165}+N_{1}\overset{k_{on,V165,N1}}{\underset{k_{off,V165N1}}{⇌}}V_{165}N_{1}$$

$$V_{165}N_{1}+R_{2}\overset{k_{c,V165N1,R2}}{\underset{k_{off,V165N1,R2}}{⇌}}R_{2}V_{165}N_{1}$$

$$V_{165}R_{2}+N_{1}\overset{k_{c,V165R2,N1}}{\underset{k_{off,V165R2,N1}}{⇌}}R_{2}V_{165}N_{1}$$

while the VEGF_121 _isoform binds to the receptors according to these reactions

$$V_{121}+R_{1}\overset{k_{on,V121,R1}}{\underset{k_{off,V121R1}}{⇌}}V_{121}R_{1}$$

$$V_{121}+R_{2}\overset{k_{on,V121,R2}}{\underset{k_{off,V121R2}}{⇌}}V_{121}R_{2}$$

$$R_{1}+N_{1}\overset{k_{c,R1,N1}}{\underset{k_{dissoc,R1N1}}{⇌}}R_{1}N_{1}$$

$$V_{121}+R_{1}N_{1}\overset{k_{on,V121,R1}}{\underset{k_{off,V121R1}}{⇌}}V_{121}R_{1}N_{1}$$

$$V_{121}R_{1}+N_{1}\overset{k_{c,R1,N1}}{\underset{k_{dissoc,R1N1}}{⇌}}V_{121}R_{1}N_{1}$$

The 10 preceding chemical reactions are governed by the following equations:

(7) $$\begin{array}{c} \frac{d[R_{1}]}{dt}=s_{R1}-k_{\mathrm{int},R1}[R_{1}]-k_{on,V165,R1}[V_{165}][R_{1}]+k_{off,V165R1}[V_{165}R_{1}]\\ -k_{on,V121,R1}[V_{121}][R_{1}]+k_{off,V121R1}[V_{121}R_{1}]\\ -k_{c,R1,N1}[N_{1}][R_{1}]+k_{dissoc,R1N1}[R_{1}N_{1}] \end{array}$$

(8) $$\begin{array}{c} \frac{d[R_{2}]}{dt}=s_{R2}-k_{\mathrm{int},R2}[R_{2}]-k_{on,V121,R2}[V_{121}][R_{2}]+k_{off,V121R2}[V_{121}R_{2}]\\ -k_{on,V165,R2}[V_{165}][R_{2}]+k_{off,V165R2}[V_{165}R_{2}]\\ -k_{c,V165N1,R2}[V_{165}N_{1}][R_{2}]+k_{off,V165N1,R2}[R_{2}V_{165}N_{1}] \end{array}$$

(9) $$\begin{array}{c} \frac{d[N_{1}]}{dt}=s_{N1}-k_{\mathrm{int},N1}[N_{1}]-k_{c,V121R1,N1}[V_{121}R_{1}][N_{1}]+k_{dissoc,R1N1}[V_{121}R_{1}N_{1}]\\ -k_{c,R1,N1}[N_{1}][R_{1}]+k_{dissoc,R1N1}[R_{1}N_{1}]-k_{on,V165,N1}[V_{165}][N_{1}]\\ -k_{off,V165N1}[V_{165}N_{1}]-k_{c,V165R2,N1}[V_{165}R_{2}][N_{1}]+k_{off,V165R2,N1}[R_{2}V_{165}N_{1}] \end{array}$$

(10) $$\begin{array}{c} \frac{d[V_{121}R_{1}]}{dt}=-k_{\mathrm{int},V121R1}[V_{121}R_{1}]+k_{on,V121,R1}[V_{121}][R_{1}]-k_{off,V121R1}[V_{121}R_{1}]\\ -k_{c,R1,N1}[V_{121}R_{1}][N_{1}]+k_{dissoc,R1N1}[V_{121}R_{1}N_{1}] \end{array}$$

(11) $$\frac{d[V_{121}R_{2}]}{dt}=-k_{\mathrm{int},V121R2}[V_{121}R_{2}]+k_{on,V121,R2}[V_{121}][R_{2}]-k_{off,V121R2}[V_{121}R_{2}]$$

(12) $$\frac{d[V_{165}R_{1}]}{dt}=-k_{\mathrm{int},V165R1}[V_{165}R_{1}]+k_{on,V165,R1}[V_{165}][R_{1}]-k_{off,V165R1}[V_{165}R_{1}]$$

(13) $$\begin{array}{c} \frac{d[V_{165}R_{2}]}{dt}=-k_{\mathrm{int},V165R2}[V_{165}R_{2}]+k_{on,V165,R2}[V_{165}][R_{2}]-k_{off,V165R2}[V_{165}R_{2}]\\ -k_{c,V165R2,N1}[V_{165}R_{2}][N_{1}]+k_{off,V165R2,N1}[R_{2}V_{165}N_{1}] \end{array}$$

(14) $$\begin{array}{c} \frac{d[V_{165}N_{1}]}{dt}=-k_{\mathrm{int},V165N1}[V_{165}N_{1}]+k_{on,V165,N1}[V_{165}][N_{1}]-k_{off,V165N1}[V_{165}N_{1}]\\ -k_{c,V165N1,R2}[V_{165}N_{1}][R_{2}]+k_{off,V165N1,R2}[R_{2}V_{165}N_{1}] \end{array}$$

(15) $$\begin{array}{c} \frac{d[R_{1}N_{1}]}{dt}=-k_{\mathrm{int},R1N1}[R_{1}N_{1}]+k_{c,R1,N1}[N_{1}][R_{1}]-k_{dissoc,R1N1}[R_{1}N_{1}]\\ -k_{on,V121,R1}[V_{121}][R_{1}N_{1}]+k_{off,V121R1}[V_{121}R_{1}N_{1}] \end{array}$$

(16) $$\begin{array}{c} \frac{d[R_{2}V_{165}N_{1}]}{dt}=-k_{\mathrm{int},V165R2N1}[R_{2}V_{165}N_{1}]+k_{c,V165R2,N1}[V_{165}R_{2}][N_{1}]\\ -k_{off,V165R2,N1}[R_{2}V_{165}N_{1}]+k_{c,V165N1,R2}[V_{165}N_{1}][R_{2}]\\ -k_{off,V165N1,R2}[R_{2}V_{165}N_{1}] \end{array}$$

(17) $$\begin{array}{c} \frac{d[V_{121}R_{1}N_{1}]}{dt}=-k_{\mathrm{int},V121R1N1}[V_{121}R_{1}N_{1}]+k_{c,V121R1,N1}[V_{121}R_{1}][N_{1}]\\ -k_{dissoc,V121N1}[V_{121}R_{1}N_{1}]+k_{on,V121R1N1}[V_{121}][R_{1}N_{1}]\\ -k_{off,V121R1N1}[V_{121}R_{1}N_{1}] \end{array}$$

### Ligands in the tissue compartments

The ligands VEGF_121 _and VEGF_165 _are secreted by the cells at a rate *q*_*V *_per unit volume of total tissue. The secretion rate is assumed to be constant. The concentrations of the ligands in tissue follow these equations:

(18) $$\begin{array}{c} \frac{d[V_{121}]}{dt}=q_{V121}-k_{on,V121,R1}[V_{121}][R_{1}]+k_{off,V121R1}[V_{121}R_{1}]-k_{on,V121,R1N1}[V_{121}][R_{1}N_{1}]\\ +k_{off,V121R1N1}[V_{121}R_{1}N_{1}]-k_{on,V121,R2}[V_{121}][R_{2}]+k_{off,V121R2}[V_{121}R_{2}] \end{array}$$

(19) $$\begin{array}{c} \frac{d[V_{165}]}{dt}=q_{V165}-k_{on,V165,MEBM}[V_{165}][M_{EBM}]+k_{off,V165,MEBM}^{N}[V_{165}M_{EBM}]\\ -k_{on,V165,MECM}[V_{165}][M_{ECM}]+k_{off,V165MECM}[V_{165}M_{ECM}]\\ -k_{on,V165,MPBM}[V_{165}][M_{PBM}]+k_{off,V165MPBM}[V_{165}M_{PBM}]\\ -k_{on,V165,R1}[V_{165}][R_{1}]+k_{off,V165R1}[V_{165}R_{1}]-k_{on,V165,R2}[V_{165}][R_{2}]\\ +k_{off,V165R2}[V_{165}R_{2}]-k_{on,V165,N1}[V_{165}][N_{1}]+k_{off,V165N1}[V_{165}N_{1}] \end{array}$$

Note that, here, the vascular permeability has yet to appear in the equations.

### Transport between the compartments

When two tissue volumes containing different amount of extracellular matrix are separated by a semipermeable membrane (endothelium), in thermodynamic equilibrium the volume concentrations of ligand would not be the same. To correctly describe this phenomenon, a volumetric correction has to be made in order to take into account that VEGF can move only in a restricted region in the interstitial space. We follow the formalism introduced by Truskey *et al*. to describe macromolecular drug distribution in tissue [18]. The extracellular matrix is a porous medium composed of proteins and polysaccharides and can deform under mechanical stress. This deformation can affect the volume accessible to the solutes. In addition, some pores are inaccessible to the freely diffusible molecules, because of their sizes or because of the tissue configuration. Finally, there is a steric exclusion of solutes near the surface of the solid phase of the extracellular matrix and the basement membranes. To reflect these properties in the model, the following parameters are introduced: Φ the partition coefficient, *ε*_*IF *_the porosity, *ε*_*IS *_the interstitial fraction, *f *the fluid fraction of the interstitial space and *K*_*AV *_the available volume fraction. These parameters are defined by and related to one another through the following equations:

$$\varepsilon_{IS}=\frac{\text{interstitial space}}{\text{total tissue volume}}$$

$$f=\frac{\text{interstitial fluid}}{\text{interstitial space}}$$

$$\Phi =\frac{\text{available fluid volume}}{\text{interstitial fluid}}$$

$$\varepsilon_{IF}=\frac{\text{interstitial fluid}}{\text{total tissue volume}}=f\times \varepsilon_{IS}$$

$$K_{AV}=\frac{\text{available fluid volume}}{\text{total tissue volume}}=\Phi \times \varepsilon_{IF}$$

The free VEGF molecules that diffuse in the interstitial space are effectively only present in the available interstitial fluid volume. In other words, the interstitial space is a part of the total tissue volume *U *and has a volume of *ε*_*IS *_× *U *. It is composed of a solid phase of volume (*ε*_*IS *_- *ε*_*IF*_) × *U *and a fluid phase of volume *ε*_*IF *_× *U *in which VEGF can circulate freely. However, given the pore size of the ECM and basement membranes and the molecular radius of VEGF, some regions are excluded because the growth factor molecules cannot access them. In conclusion, the VEGF molecules can diffuse in the available interstitial fluid volume *U*_*AV *_= *K*_*AV *_× *U *. Similarly, the free VEGF concentration in the available interstitial fluid volume, noted [*V*]_*AV*_, is related to the free VEGF concentration in the total tissue volume [*V*] by ${\left[V\right]}_{AV}=\frac{\left[V\right]}{K_{AV}}$.

Until now, all the equations have been expressed per unit volume of total tissue. However, because of volume exclusion, we now introduce explicit available interstitial volumes. We assign the subscripts *N *for healthy (or normal) tissue, *D *for diseased tissue, *B *for blood and *p *for blood plasma (available fluid volume to VEGF in the blood). Note that these subscripts are written as superscripts in the kinetic parameters for clarity reasons. The transport of free VEGF molecules from normal tissue to the blood occurs at a rate $k_{pV}^{NB}$ (units: cm/s). The term $k_{pV}^{NB}$ reads as "rate of permeability (*k*_*p*_) of VEGF (*V*) from the normal tissue compartment (*N*) to the blood (*B*)". The free VEGF molecules extravasate at a rate $k_{pV}^{BN}$ (units: cm/s). The interface between the normal tissue and the blood is the total surface of the microvessels denoted as *S*_*NB *_(units: cm^2^).

(20) $$U_{AV,N}\frac{d{\left[V\right]}_{AV,N}}{dt}=-k_{pV}^{NB}S_{NB}{\left[V\right]}_{AV,N}+k_{pV}^{BN}S_{NB}{\left[V\right]}_{p}$$

Note that, [*V*]_*AV *_× *U*_*AV *_= [*V*] × *U *in the tissue compartments and [*V*]_*p *_× *U*_*p *_= [*V*]_*B *_× *U*_*B *_in the blood compartment. Equation (20) can be rewritten in terms of moles per unit volume of total tissue

(21) $$\frac{d{\left[V\right]}_{N}}{dt}=-k_{pV}^{NB}\frac{S_{NB}}{U_{N}}\frac{{\left[V\right]}_{N}}{K_{AV,N}}+k_{pV}^{BN}\frac{S_{NB}}{U_{N}}\frac{U_{B}}{U_{p}}{\left[V\right]}_{B}$$

VEGF binding to the receptors, the extracellular matrix and the basement membranes (Eq. (18) and (19)) are added leading to the full equations governing each free isoform in the normal tissue:

(22) $$\begin{array}{c} \frac{d{\left[V_{121}\right]}_{N}}{dt}=q_{V121}^{N}-k_{on,V121,R1}^{N}{\left[V_{121}\right]}_{N}{\left[R_{1}\right]}_{N}+k_{off,V121R1}^{N}{\left[V_{121}R_{1}\right]}_{N}\\ -k_{on,V121,R1N1}^{N}{\left[V_{121}\right]}_{N}{\left[R_{1}N_{1}\right]}_{N}+k_{off,V121R1N1}^{N}{\left[V_{121}R_{1}N_{1}\right]}_{N}\\ -k_{on,V121,R2}^{N}{\left[V_{121}\right]}_{N}{\left[R_{2}\right]}_{N}+k_{off,V121R2}^{N}{\left[V_{121}R_{2}\right]}_{N}\\ -k_{pV}^{NB}\frac{S_{NB}}{U_{N}}\frac{{\left[V_{121}\right]}_{N}}{K_{AV,N}}+k_{pV}^{BN}\frac{S_{NB}}{U_{N}}\frac{U_{B}}{U_{p}}{\left[V_{121}\right]}_{B} \end{array}$$

(23) $$\begin{array}{c} \frac{d{\left[V_{165}\right]}_{N}}{dt}=q_{V165}^{N}-k_{on,V165,MEBM}^{N}{\left[V_{165}\right]}_{N}{\left[M_{EBM}\right]}_{N}+k_{off,V165,MEBM}^{N}{\left[V_{165}M_{EBM}\right]}_{N}\\ -k_{on,V165,MECM}^{N}{\left[V_{165}\right]}_{N}{\left[M_{ECM}\right]}_{N}+k_{off,V165MECM}^{N}{\left[V_{165}M_{ECM}\right]}_{N}\\ -k_{on,V165,MPBM}^{N}{\left[V_{165}\right]}_{N}{\left[M_{PBM}\right]}_{N}+k_{off,V165MPBM}^{N}{\left[V_{165}M_{PBM}\right]}_{N}\\ -k_{on,V165,R1}^{N}{\left[V_{165}\right]}_{N}{\left[R_{1}\right]}_{N}+k_{off,V165R1}^{N}{\left[V_{165}R_{1}\right]}_{N}-k_{on,V165,R2}^{N}{\left[V_{165}\right]}_{N}{\left[R_{2}\right]}_{N}\\ +k_{off,V165R2}^{N}{\left[V_{165}R_{2}\right]}_{N}-k_{on,V165,N1}^{N}{\left[V_{165}\right]}_{N}{\left[N_{1}\right]}_{N}+k_{off,V165N1}^{N}{\left[V_{165}N_{1}\right]}_{N}\\ -k_{pV}^{NB}\frac{S_{NB}}{U_{N}}\frac{{\left[V_{165}\right]}_{N}}{K_{AV,N}}+k_{pV}^{BN}\frac{S_{NB}}{U_{N}}\frac{U_{B}}{U_{p}}{\left[V_{165}\right]}_{B} \end{array}$$

Similarly, in the diseased tissue compartment, VEGF_121 _and VEGF_165 _are secreted by cells at a rate *q*_*V *_. The secretion rate is assumed constant. Free VEGF ligands can enter the blood at a rate $k_{pV}^{DB}$ and extravasate at a rate $k_{pV}^{BD}$. The interface between the diseased tissue and the blood is the total surface of the microvessels denoted as *S*_*DB*_. The equations governing each VEGF isoform read:

(24) $$\begin{array}{c} \frac{d{\left[V_{121}\right]}_{D}}{dt}=q_{V121}^{D}-k_{on,V121,R1}^{D}{\left[V_{121}\right]}_{D}{\left[R_{1}\right]}_{D}+k_{off,V121R1}^{D}{\left[V_{121}R_{1}\right]}_{D}\\ -k_{on,V121,R1N1}^{D}{\left[V_{121}\right]}_{D}{\left[R_{1}N_{1}\right]}_{D}+k_{off,V121R1N1}^{D}{\left[V_{121}R_{1}N_{1}\right]}_{D}\\ -k_{on,V121,R2}^{D}{\left[V_{121}\right]}_{D}{\left[R_{2}\right]}_{D}+k_{off,V121R2}^{D}{\left[V_{121}R_{2}\right]}_{D}\\ -k_{pV}^{DB}\frac{S_{DB}}{U_{D}}\frac{{\left[V_{121}\right]}_{D}}{K_{AV,D}}+k_{pV}^{BD}\frac{S_{DB}}{U_{D}}\frac{U_{B}}{U_{p}}{\left[V_{121}\right]}_{B} \end{array}$$

(25) $$\begin{array}{c} \frac{d{\left[V_{165}\right]}_{D}}{dt}=q_{V165}^{D}-k_{on,V165,MEBM}^{D}{\left[V_{165}\right]}_{D}{\left[M_{EBM}\right]}_{D}+k_{off,V165,MEBM}^{D}{\left[V_{165}M_{EBM}\right]}_{D}\\ -k_{on,V165,MECM}^{D}{\left[V_{165}\right]}_{D}{\left[M_{ECM}\right]}_{D}+k_{off,V165MECM}^{D}{\left[V_{165}M_{ECM}\right]}_{D}\\ -k_{on,V165,MPBM}^{D}{\left[V_{165}\right]}_{D}{\left[M_{PBM}\right]}_{D}+k_{off,V165MPBM}^{D}{\left[V_{165}M_{PBM}\right]}_{D}\\ -k_{on,V165,R1}^{D}{\left[V_{165}\right]}_{D}{\left[R_{1}\right]}_{D}+k_{off,V165R1}^{D}{\left[V_{165}R_{1}\right]}_{D}-k_{on,V165,R2}^{D}{\left[V_{165}\right]}_{D}{\left[R_{2}\right]}_{D}\\ +k_{off,V165R2}^{D}{\left[V_{165}R_{2}\right]}_{D}-k_{on,V165,N1}^{D}{\left[V_{165}\right]}_{D}{\left[N_{1}\right]}_{D}+k_{off,V165N1}^{D}{\left[V_{165}N_{1}\right]}_{D}\\ -k_{pV}^{DB}\frac{S_{DB}}{U_{D}}\frac{{\left[V_{165}\right]}_{D}}{K_{AV,D}}+k_{pV}^{BD}\frac{S_{DB}}{U_{D}}\frac{U_{B}}{U_{p}}{\left[V_{165}\right]}_{B} \end{array}$$

We assume that no receptors are present on the luminal side of the endothelial cells. Therefore, only free ligands exist in the blood compartment. Nevertheless, receptors in this compartment could be added to the model as experimental knowledge emerges and the density of receptors on the luminal side of the endothelial cells is quantified. The presence of platelets and leukocytes as potential VEGF carriers, is neglected in the current model as well as soluble VEGFR1 (sFlt) as a VEGF-neutralizing molecule. Given the complexity of the model, it is preferable to introduce additional factors one at a time and investigate their effects (a computational equivalent of knock-in or knock-out procedures). The model can be readily extended to include these factors.

Finally, in the blood compartment, VEGF is cleared at a rate *c*_*V *_(units: s^-1^) per unit volume of total blood via several organs (e.g., kidneys) that are not explicitly represented here. This process is described as a first-order reaction. Similarly to the equations for free VEGF in normal and diseased tissues, the governing equation for each free isoform in the blood is:

(26) $$U_{p}\frac{d{\left[V\right]}_{p}}{dt}=-k_{pV}^{BN}S_{NB}{\left[V\right]}_{p}+k_{pV}^{NB}S_{NB}\frac{{\left[V\right]}_{N}}{K_{AV,N}}-k_{pV}^{BD}S_{DB}{\left[V\right]}_{p}+k_{pV}^{DB}S_{DB}\frac{{\left[V\right]}_{D}}{K_{AV,D}}$$

This can be rewritten per unit volume of whole blood by:

(27) $$\frac{d{\left[V\right]}_{B}}{dt}=-k_{pV}^{BN}\frac{S_{NB}}{U_{p}}{\left[V\right]}_{B}+k_{pV}^{NB}\frac{S_{NB}}{U_{B}}\frac{{\left[V\right]}_{N}}{K_{AV,N}}-k_{pV}^{BD}\frac{S_{DB}}{U_{p}}{\left[V\right]}_{B}+k_{pV}^{DB}\frac{S_{DB}}{U_{B}}\frac{{\left[V\right]}_{D}}{K_{AV,D}}$$

Therefore, the VEGF isoforms are governed by the two following equations.

(28) $$\begin{array}{c} \frac{d{\left[V_{121}\right]}_{B}}{dt}=-c_{V121}{\left[V_{121}\right]}_{B}-k_{pV}^{BN}\frac{S_{NB}}{U_{p}}{\left[V_{121}\right]}_{B}+k_{pV}^{NB}\frac{S_{NB}}{U_{B}}\frac{{\left[V_{121}\right]}_{N}}{K_{AV,N}}\\ -k_{pV}^{BD}\frac{S_{DB}}{U_{p}}{\left[V_{121}\right]}_{B}+k_{pV}^{DB}\frac{S_{DB}}{U_{B}}\frac{{\left[V_{121}\right]}_{D}}{K_{AV,D}} \end{array}$$

(29) $$\begin{array}{c} \frac{d{\left[V_{165}\right]}_{B}}{dt}=-c_{V165}{\left[V_{121}\right]}_{B}-k_{pV}^{BN}\frac{S_{NB}}{U_{p}}{\left[V_{165}\right]}_{B}+k_{pV}^{NB}\frac{S_{NB}}{U_{B}}\frac{{\left[V_{165}\right]}_{N}}{K_{AV,N}}\\ -k_{pV}^{BD}\frac{S_{DB}}{U_{p}}{\left[V_{165}\right]}_{B}+k_{pV}^{DB}\frac{S_{DB}}{U_{B}}\frac{{\left[V_{165}\right]}_{D}}{K_{AV,D}} \end{array}$$

### Whole-body parameters

Each tissue is uniquely characterized by its biochemical, biophysical and geometrical properties as input parameters into the model. Similar to the consideration of total tissue volume compared to available interstitial fluid volume, we distinguish between the total tissue volume of the ECM, PBM and EBM, noted *U*_*ECM*_, *U*_*PBM*_, *U*_*EBM *_respectively, and the available interstitial fluid volumes of the ECM, PBM and EBM, noted *U*_*AV*, *ECM*_, *U*_*AV*, *PBM*_, *U*_*AV*, *EBM *_respectively. In terms of these variables various tissue characteristics can be calculated for the different tissue compartments, e.g., the total amount of VEGF_165 _bound to the binding sites of the ECM is equal to *U*_*AV*, *ECM *_× [*V*_165_*M*_*ECM*_]_*AV*_, where [*V*_165 _M_*ECM*_]_*AV *_is the concentration of VEGF_165 _bound to the ECM in moles per liter of available ECM fluid volume. Similarly, the total amount of VEGF_165 _sequestered by the EBM is equal to *U*_*AV*, *EBM *_× [*V*_165_*M*_*EBM*_]_*AV*_, where [*V*_165_*M*_*EBM*_]_*AV *_is the concentration of VEGF_165 _bound to the endothelial basement membrane in moles per liter of available EBM fluid volume. The conversion is given by

(30) *U*_*AV*, *EBM *_[*M*_*EBM*_]_*AV *_= [*M*_*EBM*_] *U*

(31) *U*_*AV*, *ECM *_[*M*_*ECM*_]_*AV *_= [*M*_*ECM*_] *U*

In addition, the total abluminal surface area of endothelial cells is *S*_*EC*_. The total amount of unligated VEGFR2 is [*R*_2_]* × *S*_*EC*_, where [*R*_2_]* is the number of receptors VEGFR2 per unit of endothelial surface.

(32) [*R*_2_]* *S*_*EC *_= [*R*_2_] *U*

Note that the kinetic rates can also be transformed, e.g.,

(33) *k*_*AV*, *on*_*U *= *k *_*on*_*U*_*AV*_

In the blood phase, we express all concentrations with respect to its total volume. If we designate red blood cell fractional volume or hematocrit as Ht and neglect the small volume of white blood cells and platelets, then the concentration of species in blood can be expressed in terms of plasma concentrations as follows

(34) [*V*_165_]_*p *_= [*V*_165_]_*B *_× (1 - *Ht*)

where [*V*_165_]_*p *_represents the concentration of free VEGF_165 _in the plasma.

### Numerical implementation

The model, represented by 40 ordinary differential equations (19 for each tissue compartment and 2 for the blood) and initial conditions, was implemented using Visual FORTRAN 6 software on a PC. The equations were non-dimensionalized prior to numerical solution using appropriate characteristic values and once the solutions were obtained were transformed to the original dimensional variables. Transient solutions were calculated using an adaptive step-size Runge-Kutta 5^th^-order accuracy integrative scheme. A relative error tolerance of 10^-5 ^was used. The steady state was defined when the concentrations changed by less than 1%.

## Model parameters

The parameters are summarized in Tables 1, 2, 3, 4, 5, 6.

**Table 1:** Geometric parameters for the healthy tissue (human vastus lateralis muscle)

**Skeletal muscle characteristic**	**Value**	**Unit**	**Reference**
***Muscle fibers***			
Cross sectional area of one fiber	3000	*μ*m^2^	[6]
Perimeter of one fiber	222	*μ*m	[6]

***Capillary-fiber ratio***	1.38		[37]

***Capillary density***	420	capillaries/mm^2^ tissue	[6]

***Muscle fiber density***	304	fibers/mm^2 ^tissue	[6]

***Volume fractions***			
Interstitial space	8.16%	cm^3^/cm^3 ^tissue	[38,39]
Fibers	89.98%	cm^3^/cm^3 ^tissue	[6]
Microvessels	1.86%	cm^3^/cm^3 ^tissue	[6]
*of which *vascular space	1.4%	cm^3^/cm^3 ^tissue	[40]

***Microvessels***			
Internal diameter of microvessel	6.56	*μ*m	[6]
Thickness of endothelial cell	0.5	*μ*m	[41]
External diameter of microvessel	7.56	*μ*m	[6]
Cross sectional area of one microvessel	45	*μ*m^2^	[6]
Perimeter of one microvessel	26	*μ*m	[6]

***Surface areas***			
Muscle fibers	664	cm^2^/cm^3 ^tissue	[6]
Microvessels	108	cm^2^/cm^3 ^tissue	[6]

***Basement membranes (BM)***			
Thickness of muscle fiber BM	24	nm	[42]
Basement membrane volume (muscle fiber)	0.00159	cm^3^/cm^3 ^tissue	Calculated
*of which *available to VEGF	0.000307	cm^3^/cm^3 ^tissue	Calculated
Thickness of microvessel BM	43	nm	[42]
Basement membrane volume (microvessel)	0.00045	cm^3^/cm^3 ^tissue	Calculated
*of which *available to VEGF	0.000087	cm^3^/cm^3 ^tissue	Calculated
Extracellular matrix volume	0.07951	cm^3^/cm^3 ^tissue	Calculated
*of which *available to VEGF	0.061987	cm^3^/cm^3 ^tissue	Calculated

***Skeletal muscle nuclear domain (SMND) surface area***	1850	*μ*m^2^	[6]

**Table 2:** Kinetic parameters of VEGF in the healthy tissue (human vastus lateralis muscle)

	**Measured parameters**		**Tissue parameters**	
	**Value**	**Unit**	**Value**	**Unit**
***VEGF binding to VEGFR1***				
k_on_	3 10^7^	M^-1 ^s^-1^	4.8 10^-1^	(pmol/cm^3 ^tissue)^-1 ^s^-1^
k_off_	10^-3^	s^-1^		
K_d_	33	pM	2.0 10^-3^	pmol/cm^3 ^tissue

***VEGF binding to VEGFR2***				
k_on_	10^7^	M^-1 ^s^-1^	1.6 10^-1^	(pmol/cm^3 ^tissue)^-1 ^s^-1^
k_off_	10^-3^	s^-1^		
K_d_	100	pM	6.4 10^-3^	pmol/cm^3 ^tissue

***VEGF165 binding to NRP1***				
k_on_	3.2 10^6^	M^-1 ^s^-1^	5.1 10^-2^	(pmol/cm^3 ^tissue)^-1 ^s^-1^
k_off_	10^-3^	s^-1^		
K_d_	312	pM	2.0 10^-2^	pmol/cm^3 ^tissue

***VEGF165 binding to GAGs***				
k_on_	4.2 10^5^	M^-1 ^s^-1^	6.7 10^-3^	(pmol/cm^3 ^tissue)^-1 ^s^-1^
k_off_	10^-2^	s^-1^		
K_d_	24	nM	1.5	pmol/cm^3 ^tissue

***Coupling of NRP1 & VEGFR2***				
k_cV165R2, N1_	3.1 10^13^	(mol/cm^2^)^-1 ^s^-1^	2.8 10^-1^	(pmol/cm^3 ^tissue)^-1 ^s^-1^
k_offV165R2,N1_	10^-3^	s^-1^		
k_cV165N1,R2_	10^14^	(mol/cm^2^)^-1 ^s^-1^	9.2 10^-1^	(pmol/cm^3 ^tissue)^-1 ^s^-1^
k_offV165N1,R2_	10^-3^	s^-1^		

***VEGFR1 coupling to NRP1***				
k_cR1,N1_	10^14^	(mol/cm^2^)^-1 ^s^-1^	9.2 10^-1^	(pmol/cm^3 ^tissue)^-1 ^s^-1^
k_dissocR1,N_	10^-2^	s^-1^		

***VEGFR internalization***				
k_int,R_	2.8 10^-4^	s^-1^		
k_int,C_	2.8 10^-4^	s^-1^		

The derivation of these parameters is found in [6]. In this table, 6.24 10^7 ^(pmol/cm^3 ^tissue)/M and 1.09 10^14 ^(pmol/cm^3 ^tissue)/(mol/cm^2 ^EC). Here, M = moles/liter available interstitial fluid volume.

**Table 3:** VEGF concentration and receptor densities for the healthy tissue (human vastus lateralis)

	**Measured parameter**		**Tissue model**	
	**Value**	**Unit**	**Value**	**Unit**
***Free VEGF concentration***				
Human vastus lateralis, rest	1	pM	6.2 10^-5^	pmol/cm^3 ^tissue

***Total VEGF tissue concentration***				
Human vastus lateralis, rest	1–2	pg/*μ*g protein	3.4–6.9	pmol/cm^3 ^tissue

***VEGFR1 tissue concentration***				
Human vastus lateralis, rest	1.6–1.8	pg/*μ*g protein	1.1–1.2	pmol/cm^3 ^tissue
			60,000–68,000	#/EC

***VEGFR2 tissue concentration***				
Human vastus lateralis, rest	0.33–0.5	pg/*μ*g protein	0.24–0.34	pmol/cm^3 ^tissue
			13,000–19,000	#/EC

***NRP1 tissue concentration***				
			0.018–1.8	pmol/cm^3 ^tissue
			1,000–100,000	#/EC

***ECM binding site density***				
ECM	0.75	*μ*M	46	pmol/cm^3 ^tissue
Vessel BM	13	*μ*M	1	pmol/cm^3 ^tissue
Myocyte BM	13	*μ*M	4	pmol/cm^3 ^tissue

The conversion of receptor densities to tissue concentrations is based on the relationship mentioned in table 2, and the surface area of an endothelial cell, 1000 *μ*m^2^. VEGF concentration is normalized based on the entire interstitial space, since it diffuses throughout: 6.2 10^7 ^(pmol/cm^3 ^tissue)/M (here, M = moles/liter interstitial space). VEGF binding sites in the ECM and BMs are based on those volumes: 6.2 10^7 ^(pmol/cm^3 ^tissue)/M(ECM), 5.7 10^4 ^(pmol/cm^3 ^tissue)/M(EBM), 3.1 10^5 ^(pmol/cm^3 ^tissue)/M (MBM). For example, M(EBM) = moles/liter endothelial basement membrane. Conversions from pg/mg protein are based on 155 mg protein/g of tissue and 45 kDa VEGF, 210 kDa VEGFR1, 240 kDa VEGFR2 [6].

**Table 4:** Geometric parameters for the breast tumor

	**Value**	**Unit**	**Reference**
***Cancer cells***			
Tumor cells external diameter	17	*μ*m	[43]
Volume of one cell	2572	*μ*m^3^	[4]
Surface area of one cell	997	*μ*m^2^	[4]

***Microvessels***			
Average luminal diameter	10.3	*μ*m	[44,45]
Endothelial cell thickness	0.5	*μ*m	[4]
Average external diameter	11.3	*μ*m	[4]
Cross sectional area of one vessel	100.3	*μ*m^2^	[4]
Perimeter of one vessel	43.7	*μ*m	[4]

***Volume fractions***			
Interstitial space	61.1%	cm^3^/cm^3 ^tissue	[46,47]
Cancer cells	37%	cm^3^/cm^3 ^tissue	[4]
Microvessels	2.4%	cm^3^/cm^3 ^tissue	[4]
*of which *intravascular space	2%		[4]

***Surface areas***			
Tumor cells	1416	cm^2^/cm^3 ^tissue	[4]
Microvessels	105	cm^2^/cm^3 ^tissue	[4]

***Basement membranes (BM)***			
Thickness of tumor cell BM	30	nm	[4]
Basement membrane volume (tumor cells)	0.00388	cm^3^/cm^3 ^tissue	Calculated
*of which *available to VEGF	0.002446	cm^3^/cm^3 ^tissue	Calculated
Thickness of microvessel BM	50	nm	[4]
Basement membrane volume (microvessels)	0.00043	cm^3^/cm^3 ^tissue	Calculated
*of which *available to VEGF	0.000270	cm^3^/cm^3 ^tissue	Calculated
Extracellular matrix volume	0.6062	cm^3^/cm^3 ^tissue	Calculated
*of which *available to VEGF	0.519308	cm^3^/cm^3 ^tissue	Calculated

**Table 5:** Kinetic parameters of VEGF in tumor (breast cancer)

	**Measured parameters**		**Tissue parameters**	
	**Value**	**Unit**	**Value**	**Unit**
***VEGF binding to VEGFR1***				
k_on_	3 10^7^	M^-1 ^s^-1^	5.8 10^-2^	(pmol/cm^3 ^tissue)^-1 ^s^-1^
k_off_	10^-3^	s^-1^		
K_d_	33	pM	1.7 10^-2^	pmol/cm^3 ^tissue

***VEGF binding to VEGFR2***				
k_on_	10^7^	M^-1 ^s^-1^	1.9 10^-2^	(pmol/cm^3 ^tissue)^-1 ^s^-1^
k_off_	10^-3^	s^-1^		
K_d_	100	pM	5.2 10^-2^	pmol/cm^3 ^tissue

***VEGF165 binding to NRP1***				
k_on_	3.2 10^6^	M^-1 ^s^-1^	6.1 10^-3^	(pmol/cm^3 ^tissue)^-1 ^s^-1^
k_off_	10^-3^	s^-1^		
K_d_	312	pM	1.6 10^-1^	pmol/cm^3 ^tissue

***VEGF165 binding to GAGs***				
k_on_	4.2 10^5^	M^-1 ^s^-1^	8.1 10^-3^	(pmol/cm^3 ^tissue)^-1 ^s^-1^
k_off_	10^-2^	s^-1^		
K_d_	24	nM	12.5	pmol/cm^3 ^tissue

***Coupling of NRP1 & VEGFR2***				
k_cV165R2,N1_	3.1 10^13^	(mol/cm^2^)^-1 ^s^-1^	2.9 10^11^	(pmol/cm^3 ^tissue)^-1 ^s^-1^
k_offV165R2,N1_	10^-3^	s^-1^		
k_cV165N1,R2_	10^14^	(mol/cm^2^)^-1 ^s^-1^	9.5 10^11^	(pmol/cm^3 ^tissue)^-1 ^s^-1^
k_offV165N1,R2_	10^-3^	s^-1^		

***VEGFR1 coupling to NRP1***				
k_cR1,N1_	10^14^	(mol/cm^2^)^-1 ^s^-1^	9.5 10^11^	(pmol/cm^3 ^tissue)^-1 ^s^-1^
k_dissocR1,N1_	10^-2^	s^-1^		

***VEGFR internalization***				
k_int,R_	2.8 10^-4^	s^-1^		
k_int,C_	2.8 10^-4^	s^-1^		

The derivation of these parameters can be found in [4]. In this table, 5.22 10^8 ^(pmol/cm^3^ tissue)/M and 1.05 10^14 ^(pmol/cm^3^ tissue)/(mol/cm^2^ EC). Here, M = moles/liter interstitial space.

**Table 6:** VEGF concentration and receptor densities for the tumor (breast cancer)

	**Measured parameter**		**Tissue model**	
	**Value**	**Unit**	**Value**	**Unit**
***Free VEGF concentration***				
Breast cancer	0.5–1.5	pM	2.6–7.83 10^-4^	pmol/cm^3 ^tissue

***Total tumor VEGF***				
Breast cancer	13	pg/mg tumor	0.3	pmol/cm^3 ^tissue

***VEGFR1 tissue concentration***				
	10^2^–10^5^	#/EC	1.7 10^-3^–1.7 10^0^	pmol/cm^3 ^tissue

***VEGFR2 tissue concentration***				
	10^4^	#/EC	1.7 10^-1^	pmol/cm^3 ^tissue

***NRP1 tissue concentration***				
	10^3^–10^6^	#/EC	1.7 10^-2^-1.7 10^1^	pmol/cm^3 ^tissue

***ECM binding site density***				
ECM	0.75	*μ*M	389	pmol/cm^3 ^tissue
Vessel BM	13	*μ*M	4	pmol/cm^3 ^tissue
Tumor cell BM	13	*μ*M	32	pmol/cm^3 ^tissue

The conversion of receptor densities to tissue concentrations is based on the relationship mentioned in table 5, and the surface area of an endothelial cell, 1000 *μ*m^2^. VEGF concentration is normalized based on the entire interstitial space, since it diffuses throughout: 5.2 10^8 ^(pmol/cm^3 ^tissue)/M (here, M = moles/liter interstitial space). VEGF binding sites in the ECM and BMs are based on those volumes: 5.2 10^8 ^(pmol/cm^3 ^tissue)/M(ECM), 2.7 10^5 ^(pmol/cm^3 ^tissue)/M(EBM), 2.5 10^6 ^(pmol/cm^3 ^tissue)/M(PBM) [4].

### Blood

We consider a human subject of 70 kg. To calculate the typical plasma and total blood volumes in a 70-kg human being, we used a study of ninety healthy subjects by Gibson and Evans [19]. The volumes were plotted against the weight of the volunteer. Linear regression was performed for males and females and both volumes were determined for 70 kg. We therefore consider 5.154 liters of total blood including 2.920 liters of blood plasma, which constitute 56.7% of total blood volume.

### Normal tissue

The volume of the normal tissue is the volume corresponding to a 70-kg subject with a vastus lateralis skeletal muscle density of 1.06 g/cm^3^, less 5154 cm^3 ^of whole blood (mass of blood is 5.164 kg for whole blood density of 1,002 g/L [20]). The parameters characterizing the healthy tissue (skeletal muscle) are summarized in Tables 1, 2, 3 and the properties and characteristics of the skeletal muscle have been described in [6]. However, a few adjustments were made. The interstitial space is composed of 14.175% of collagen (in the ECM and basement membranes) [21]. This content is not accessible to the VEGF molecules and thus does not account for the available interstitial fluid volume. Interstitial fluid of muscle has been measured at 7% of tissue volume [6], thus the total interstitial space is 8.16% (Table 1). The pore sizes of the basement membranes and the extracellular matrix in the skeletal muscle are estimated at 7 nm [20] and 66 nm [19] respectively. For a molecular weight of 45 kDa (VEGF molecule), this corresponds to a partition coefficient Φ of 0.35 and a *K*_*AV *_of 0.0245 for the PBM and EBM [22] and a partition coefficient of Φ of 0.9 and a *K*_*AV *_of 0.063 for the ECM [22]. The available volumes for VEGF transport in the extracellular matrix, parenchymal basement membrane and endothelial basement membrane are therefore 0.061987, 0.000307, and 0.000087 cm^3^/cm^3 ^tissue, respectively (Table 1).

For the VEGF receptors, we assume that, at any instant, the insertion of receptors equals the internalization. As a result, the number of total (free and bound) receptors is conserved. This condition also applies to the diseased tissue. Regulation of VEGF receptors represents an important poorly explored area; more complex receptor dynamics can be considered in the model as experimental information becomes available.

### Diseased tissue

As an example of diseased tissue, we consider a 4 cm-diameter breast tumor. For this tissue, we use parameters reported in a previous one-compartment model [4]. Assuming the tumor to be a sphere, the volume of the diseased compartment is 33 cm^3^. The parameters are summarized in Tables 4, 5, 6 and the properties and characteristics of the tumor tissue have been described in [4]. The 5% collagen content increases the interstitial space from 58.0% [4] to 61.1%. In breast tumor, the pore size of the endothelial basement membrane has been measured to be several hundred nanometers (200 nm [23] and between 400–600 nm [24]) which corresponds to a partition coefficient of 0.9 [22]. It was shown that the basement membranes and the ECM are similar and difficult to distinguish in the context of mammary tumor [25]. Therefore, a partition coefficient of 0.9 was also adopted for the ECM (same partition coefficient as in the vastus lateralis skeletal muscle) and the PBM. This corresponds to a *K*_*AV *_of 0.522 for the three regions [22]. The available interstitial fluid volumes for VEGF transport in the extracellular matrix, parenchymal and endothelial basement membranes are therefore 0.519308, 0.002446, and 0.000270 cm^3^/cm^3 ^tissue, respectively.

### Permeability between the normal tissue and the blood

To determine the permeability between the normal tissue and the capillaries, we first determine the Stokes-Einstein radius for a VEGF molecule. The molecular weight of VEGF_165 _isoform is approximately 45 kDa. The Stokes-Einstein radius *a*_*e *_(in Å) is calculated by the formula for globular molecules given in Venturoli and Rippe [26]: *a*_*e *_= 0.483 × (*MW*)^0.386 ^. For 45 kDa, we thus find a Stokes-Einstein radius of 30.2 Å. With this effective molecular radius, it is then possible to determine the permeability-surface area product [27] as 2.5 × 10^-4 ^mL/s × 100 g. With a surface area of 70 cm^2^/g [27], we deduce that the permeability between the normal tissue and the blood is 4.3 × 10^-8 ^cm/s. A similar value was found using the permeability-surface area product using Schmittmann and Rohr's study [28]. Because there is paucity of experimental data on VEGF-dependence of macromolecular permeability [29,30], we consider a permeability range from 4 × 10^-9 ^to 4 × 10^-6 ^cm/s for sensitivity analyses; this range includes the 2–3 fold VEGF-dependent increase in permeability reported in [29,30]. When the permeability between the normal tissue and the blood is fixed, the value 4 × 10^-8 ^cm/s is selected. The VEGF transport is assumed to be passive.

### Permeability between the diseased tissue and the blood

For the permeability between the tumor and the blood, we note that ovalbumin and the VEGF homodimer have a similar molecular weight (45 kDa). In human tumor xenografts, the microvascular permeability for ovalbumin, a globular molecule, was measured to be 5.77 × 10^-7 ^cm/s [24]. Studies of tumor microvascular permeability for macromolecules in vivo also provide useful insights [31]. However, most of these experiments use dextrans that are linear molecules contrary to VEGF. This difference is crucial for permeability purposes. To resolve this issue, the relationship between the Stokes-Einstein radius and the permeability is usually preferred. For a Stokes-Einstein radius of about 30.2 Å (30.8 Å for ovalbumin), the permeability is around 6 × 10^-7 ^cm/s [24]. Therefore, a range from 4 × 10^-8 ^to 4 × 10^-5 ^cm/s is chosen for the vascular permeability to VEGF in tumor when the sensitivity analysis is performed since little data on VEGF-dependence to macromolecule permeability is available [29,30]. When the tumor/blood permeability is fixed at a specific value, 4 × 10^-7 ^cm/s is assumed.

### Summary of experimental measurements of VEGF concentration

A meta-analysis was performed by Kut *et al*. [11]. In breast cancer, plasma VEGF levels were 2 to 10 times higher that those in healthy subjects (37 – 310 *vs*. 27 – 30 pg·mL^-1^). In prostate cancer, plasma VEGF levels were 3 to 10 times higher and in colorectal cancer about two times higher that those in healthy controls.

For a VEGF molecular weight of 45 kDa, the plasma levels in breast cancer patients correspond to 0.82 – 6.89 pM while they are in the range of 0.59 – 0.65 pM for healthy subjects. In our study, we therefore assume a VEGF plasma level of around 1 pM in healthy subjects and several-fold higher in breast cancer patients. The VEGF secretion rates are then calculated to match the observed plasma level.

## Results

In all simulations, unless specified otherwise, the vascular permeability of healthy tissue is 4 × 10^-8 ^cm/s, the VEGF plasma clearance 0.0206 min^-1 ^[32], the VEGF_165 _secretion rate 0.102 molecule/cell/s in the normal tissue, the VEGF isoform expression ratio VEGF_165_:VEGF_121 _92%:8% [33] and the density of VEGFR1, VEGFR2 and NRP1 is 10,000 molecules/endothelial cell. These parameter values are based on currently available experimental data and they can be altered as additional data become available. In many cases, we systematically explore the sensitivity of the results to the parameter variation. VEGF represents the total VEGF, i.e., VEGF_121 _and VEGF_165_. This means that the free VEGF concentration corresponds to the sum of the free VEGF_165 _and the free VEGF_121 _concentrations. Similarly, the VEGF secretion rate represents the sum of the secretion rates of the two VEGF isoforms.

### Healthy subject (no diseased compartment)

#### Without VEGF clearance, steady-state blood and tissue concentrations are the same

**Figure 2:**
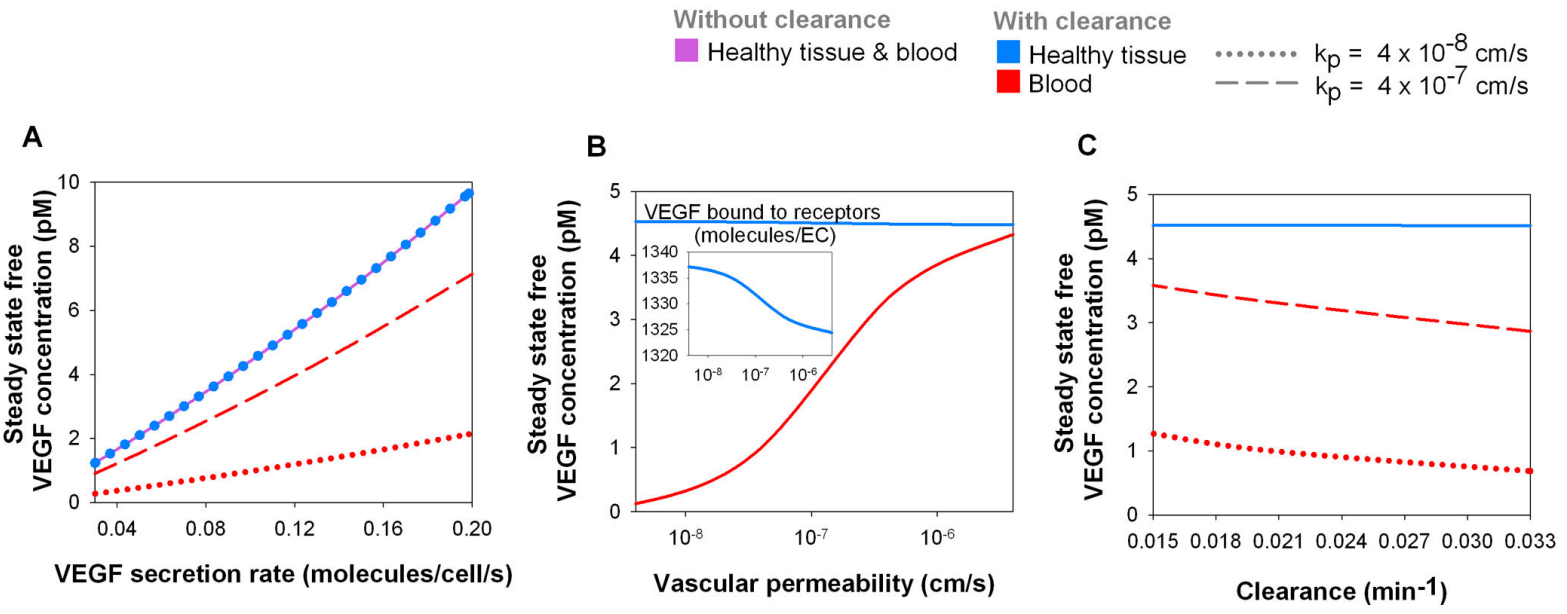
**Blood VEGF, but not tissue VEGF concentration, is dependent on VEGF clearance and vascular permeability**. The diseased compartment is not included here. **A**, Tissue and blood VEGF concentrations increase with VEGF secretion rate. The profile is approximately linear in each case. In the absence of VEGF clearance from the blood, the steady-state free VEGF concentration is the same in the tissue and in the blood (purple line). With a clearance rate of *c*_*V *_= 0.0206 min^-1^, the blood concentration (red line) is lower than that of the normal tissue (blue dots), which is unchanged by the clearance. Increase in tissue-blood permeability from *k*_*p *_= 4 × 10^-8 ^cm/s (dotted line) to 4 × 10^-7 ^cm/s (dashed line), raises the blood VEGF concentration but not the tissue concentration. **B**, Increasing transcapillary permeability increases the blood VEGF concentration at steady state. VEGF_165 _secretion rate *q *= 0.102 molecule/cell/s, clearance rate *c*_*V *_= 0.0206 min^-1 ^[28]. **C**, Increased clearance rate of VEGF from the blood decreases blood concentration of VEGF, without decreasing tissue VEGF. VEGF_165 _secretion rate *q *= 0.102 molecule/cell/s, vascular permeabilities *k*_*p *_= 4 × 10^-8 ^cm/s (dotted line) and 4 × 10^-7 ^cm/s (dashed line). For all simulations, VEGFR1 = 10,000, VEGFR2 = 10,000 and NRP1 = 10,000 molecules/endothelial cell.

A range from 0.02 to 0.20 molecule/cell/s was tested for VEGF_165 _secretion rate. In the absence of plasma clearance, the free VEGF concentration in both compartments is close to directly proportional (R^2 ^= 0.9973) to the VEGF secretion rate in the normal tissue for the range we tested (Figure 2A). In the absence of plasma clearance, the steady-state total VEGF concentration in the blood plasma equals that in the available interstitial fluid of the healthy tissue (i.e., diffusible VEGF contained in the accessible part of the fluid in the healthy tissue). This is in agreement with *V *equation (26) which, at steady state, becomes ${\left[V\right]}_{p}=\frac{{\left[V\right]}_{N}}{K_{AV,N}}={\left[V\right]}_{AV,N}$. If 1 pM (1 pmole/L of available interstitial fluid) of free VEGF concentration is present, at steady state, in the normal tissue available interstitial fluid, a VEGF concentration in the blood will also be 1 pM (1 pmole/L of blood plasma).

In the absence of plasma clearance, the steady-state free VEGF concentrations in both compartments are independent of the permeability value as long as this value is non-zero (data not shown). This is because the permeability affects the dynamics of the system by regulating how fast the compartments reach the steady state but not the steady-state free VEGF concentrations themselves. Looking at equations (28) and (29), in the absence of the tumor compartment and of plasma clearance, the steady-state free VEGF concentration does not depend on the permeability between the healthy tissue and the blood since $k_{pV}^{NB}=k_{pV}^{BN}$ is assumed. The equation reads ${\left[V\right]}_{B}=\frac{U_{p}}{U_{B}}\frac{{\left[V\right]}_{N}}{K_{AV,N}}$. In other words, the relationship ${\left[V\right]}_{p}=\frac{{\left[V\right]}_{N}}{K_{AV,N}}={\left[V\right]}_{AV,N}$ holds independently of the permeability value as long as $k_{pV}^{NB}=k_{pV}^{BN}$ holds true.

#### Plasma clearance reduces blood VEGF concentration

The VEGF half-life in plasma has been shown to be 33.7 ± 13.7 min [32]. To explore the effect of the half-life, the plasma clearance rate *c*_*V *_was chosen between 0.0146 and 0.0347 min^-1^.

With the introduction of a clearance rate *c*_*V *_of 0.0206 min^-1 ^(corresponding to a VEGF half-life of 33.7 min), the steady-state concentration of free VEGF in plasma becomes 4.5 times lower than in available interstitial fluid at a vascular permeability of 4 × 10^-8 ^cm/s and 1.3 times lower at a permeability of 4 × 10^-7 ^cm/s (Figure 2A). The dependence on VEGF secretion rate is approximately linear for the range studied. Unlike free VEGF concentration in normal tissue, free VEGF concentration in the blood is strongly dependent on the vascular permeability. This is because, at steady state, the permeability coefficients do not cancel out in the equations (28) and (29).

$$\left(c_{V}\frac{1}{k_{pV}^{BN}}\frac{U_{B}}{S_{NB}}+\frac{U_{B}}{U_{p}}\right){\left[V\right]}_{B}=\frac{{\left[V\right]}_{N}}{K_{AV,N}}$$

Next, the clearance rate is fixed at 0.0206 min^-1^. The VEGF_165 _secretion rate in the healthy tissue is chosen to be 0.102 molecule/cell/s so that, at steady state, there is 1 pM of free VEGF in the blood for a vascular permeability of 4 × 10^-8 ^cm/s. We investigate the dependence on permeability in a range 4 × 10^-9 ^to 4 × 10^-6 ^cm/s. At steady state, the free VEGF concentration in the available interstitial fluid in the normal tissue is around 4.5 pM independent of the permeability (Figure 2B). At very low permeability (4 × 10^-9 ^to 4 × 10^-8 ^cm/s), very few VEGF molecules secreted in the normal tissue enter the blood compartment. Because the clearance is directly proportional to the VEGF concentration in the blood plasma (Equations (28) and (29)), a few molecules of VEGF are cleared from the blood. Therefore, the internalization is very high and accounts for most of the loss of VEGF in the normal tissue. At very high permeability (4 × 10^-7 ^to 4 × 10^-6 ^cm/s), a high concentration of free VEGF enters the blood compartment and is cleared. The internalization is smaller and the plasma clearance is responsible for most of the loss of VEGF. In the physiological range (4 × 10^-8 ^to 4 × 10^-7 ^cm/s), the free VEGF concentration in the blood plasma is roughly proportional to the permeability between the two compartments. In the healthy tissue, however, the change in the net flow (expressed in moles of VEGF per unit time) from the healthy tissue to the blood due to permeability changes is balanced by slight changes in the binding to and the internalization of VEGF by the receptors; thus, the healthy tissue VEGF concentration vary slightly with the vascular permeability.

Next, we explore the sensitivity of the results to plasma clearance in a range between 0.0146 and 0.0347 min^-1^. The internalization of VEGF by the receptors in healthy tissue regulates VEGF concentration in the compartment (figure 2C). In the blood, however, the VEGF molecules can accumulate if the clearance rate is decreased (i.e., longer half-life).

#### Transient effects of acute exercise

Given the clearance rate (0.0206 min^-1 ^[32]), vascular permeability (4 × 10^-8 ^cm/s) and VEGF_165_:VEGF_121 _expression ratio (92%:8%), the model predicts that a VEGF_165 _secretion rate of 0.102 molecule/cell/s is necessary to achieve a free VEGF plasma level of 1 pM, as reported under physiological conditions [11]. This leads to a free VEGF concentration in the normal tissue of approximately 4.5 pM.

**Figure 3:**
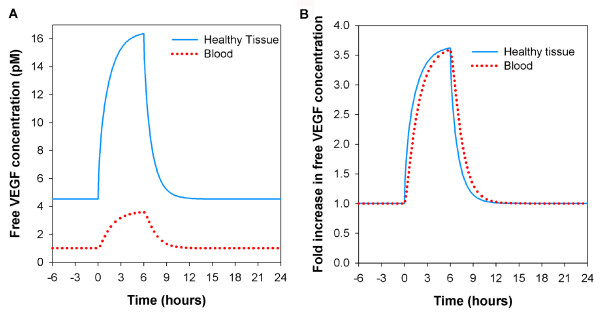
**VEGF distribution during exercise training**. The diseased compartment is not included here. Basal VEGF_165 _secretion rate *q *= 0.102 molecule/cell/s; clearance rate *c*_*V *_= 0.0206 min^-1 ^[28]; vascular permeability for VEGF *k*_*p *_= 4 × 10^-8 ^cm/s; VEGFR1 = 10,000, VEGFR2 = 10,000, NRP1 = 10,000 molecules/endothelial cell. Simulation of a 3-hour two-legged knee extension that has been shown to upregulate VEGF mRNA by about 3.5 fold for at least 6 hours [13]. **A**, Free VEGF concentration in tissue and blood. Six hours after upregulated secretion ceases, the free VEGF concentration returns to baseline. **B**, Fold increase in free VEGF concentration. The fold increase of VEGF level in the blood is equal to that in the interstitial available fluid. There is a lag of response in the blood compartment.

To study the VEGF transient effects, we simulate a physical exercise experiment. Jensen *et al*. have shown that a 3-hour two-legged knee extension upregulates VEGF mRNA by about 3.5 fold for at least 6 hours [13]. VEGF mRNA levels return to baseline between 6 and 24 hours [13]. We assume a direct correlation between mRNA and VEGF protein level. Figure 3A shows the transient effect on free VEGF concentration in available interstitial fluid and in blood plasma. The free VEGF concentrations return to baseline within 6 hours of cessation of the secretion upregulation (Figure 3A). It is interesting to note that the fold increase in blood and tissue concentrations are the same (Figure 3B). However, there is a time lag between the blood and normal tissue; in transition periods, VEGF levels measured in plasma and tissue samples could be significantly different. Higher permeability would decrease this time lag (data not shown). This provides important insights on monitoring VEGF protein levels in the case of exercise training. This simulation demonstrates that VEGF tissue concentration can increase several-fold during prolonged exercise, thus providing a stimulus for exercise-induced angiogenesis.

### Cancer patient (normal tissue, blood and tumor compartments)

In this simulation, a 4-cm diameter tumor located in the breast is represented by the diseased tissue compartment. The characteristics of this new compartment are presented in tables 4, 5, 6.

The compilation of the VEGF levels in healthy subjects and cancer patients [11] has revealed that cancer patients show, on average, a several-fold increase in their free VEGF blood plasma levels. This part of the study investigates the possible origins of this increase.

#### Blood and normal tissue VEGF levels are not significantly sensitive to tumor VEGF secretion

**Figure 4:**
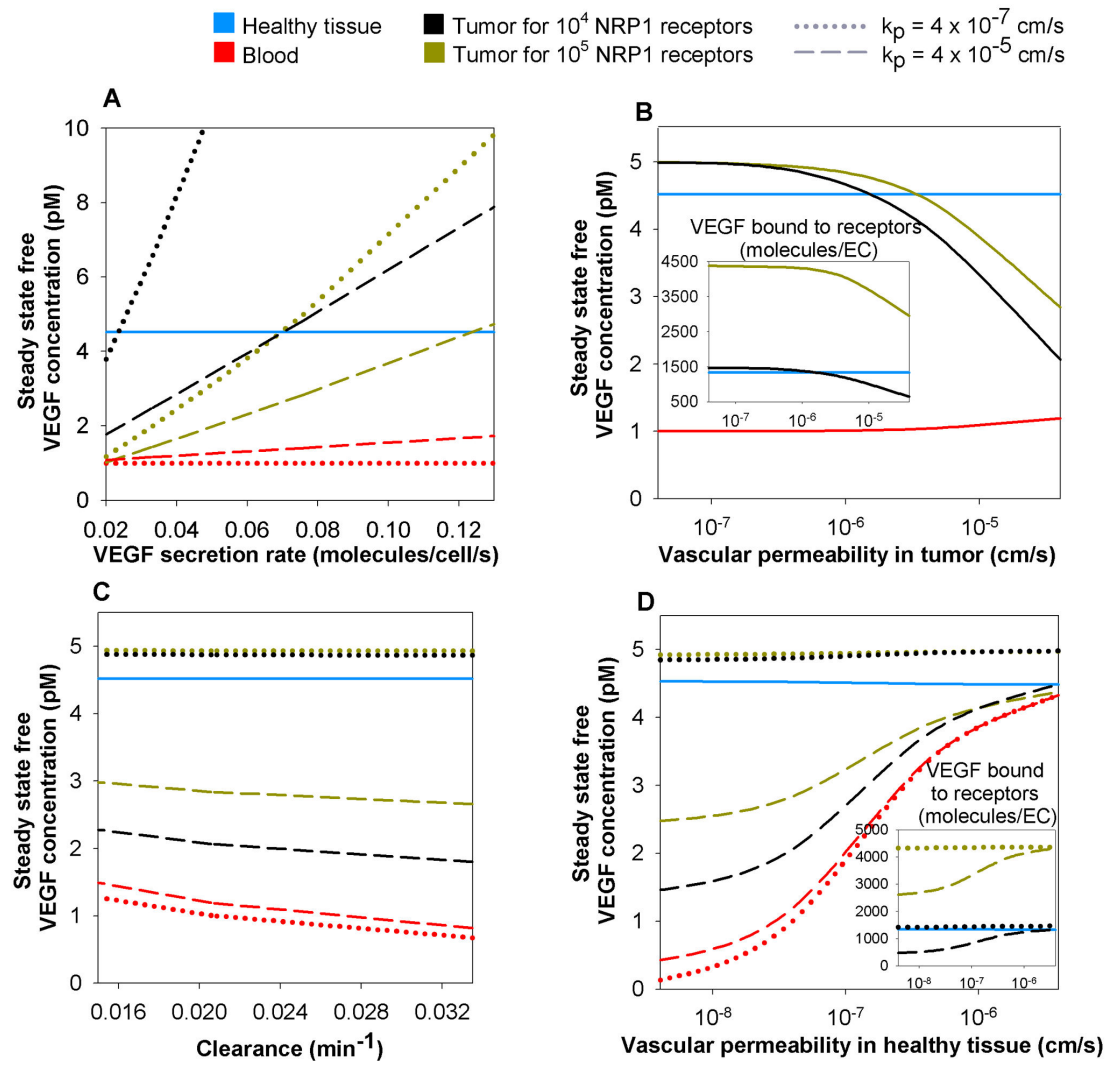
**Whole-body changes in response to VEGF secretion by a tumor**. The diseased compartment represents a 4-cm diameter tumor. Vascular permeability of the healthy tissue, $k_{p}^{N}$ = 4 × 10^-8 ^cm/s; VEGF plasma clearance *c*_*V *_= 0.0206 min^-1 ^[28]; VEGFR1 = 10,000 and VEGFR2 = 10,000 molecules/endothelial cell; NRP1 = 10,000 molecules/endothelial cell in the healthy tissue; VEGF_165 _secretion rate in healthy tissue *q*_*N *_= 0.102 molecule/cell/s; tumor VEGF_165 _secretion rate *q*_*D *_= 0.076 or 0.025 molecule/cell/s for 10,000 (black lines) or 100,000 (dark yellow lines) NRP1 in tumor respectively. The normal tissue VEGF level is insensitive to the variation of each of the parameters considered here. **A**, The free VEGF concentration in the tumor and in the blood are approximately linearly dependent on tumor VEGF secretion rate. The tumor VEGF level decreases while the blood VEGF level increases when increasing the vascular permeability in tumor from $k_{p}^{D}$ = 4 × 10^-7 ^cm/s (dotted lines) to 4 × 10^-5 ^cm/s (dashed lines). **B**, Increasing vascular permeability in tumor decreases free VEGF in the tumor and slightly increases blood VEGF. Increasing the NRP1 receptor density increases the VEGF level in tumor for vascular permeability in tumor higher than 4 × 10^-7 ^cm/s. **C**, Decreasing the clearance rate of VEGF increases free VEGF in the blood and tumor. Increasing the density of NRP1 receptors in the tumor has an effect only for higher permeability $k_{p}^{D}$ = 4 × 10^-5 ^cm/s, drastically lowering free VEGF concentration in the tumor. **D**, Increasing vascular permeability in the healthy tissue results in increased free VEGF in the blood and tumor by several-fold. Vascular permeability in the tumor $k_{p}^{D}$ = 4 × 10^-7 ^cm/s.

We performed a sensitivity analysis on the tumor VEGF secretion rate. For the selected parameters we found that regardless of the vascular permeability in the tumor, free VEGF concentration in available interstitial fluid in the normal tissue is insensitive to the VEGF secretion rate in the tumor and to the vascular permeability of the tumor (Figures 4A). This qualitative behavior is independent of the density of the NRP1 in the tumor. At a vascular permeability in the tumor of 4 × 10^-7 ^cm/s (dotted lines), the free VEGF level in the available tumor interstitial fluid is approximately proportional to the secretion rate of VEGF in the tumor while the VEGF concentration in the blood plasma is rather insensitive to the change in the tumor VEGF secretion. When increasing the permeability by two orders of magnitude (4 × 10^-5 ^cm/s, dashed lines), more VEGF molecules secreted from the tumor enter the blood. This results in an increase of VEGF level in the blood plasma and a decrease of VEGF level in the tumor for a given VEGF secretion rate in tumor. However, even at a high VEGF secretion rate in the tumor, the plasma VEGF concentration increases by less that 50%. Thus, an increase in the VEGF secretion rate alone cannot explain the several-fold increase reported for cancer patients [11], at least for the selected parameters of the model. For a given secretion rate in tumor, our calculations show that for the plasma VEGF level to double, the tumor size would have to increase approximately to 25-cm diameter.

We now set the tumor VEGF_165 _secretion rate at 0.076 or 0.025 molecule/cell/s for 10,000 and 100,000 NRP1 in the tumor respectively, so that, at a vascular permeability in tumor of 4 × 10^-8 ^cm/s (which corresponds to the vascular permeability of healthy tissue), the steady-state free VEGF concentration in the available interstitial fluid in the tumor corresponds to that in the available interstitial fluid in the normal tissue (~4.5 pM).

#### Increasing vascular permeability of tumor decreases tumor VEGF levels

We then perform a sensitivity analysis on the vascular permeability in the tumor (range from 4 × 10^-8 ^to 4 × 10^-5 ^cm/s). We find that the free VEGF concentration in the normal tissue remains constant independently of the tumor vascular permeability (Figure 4B). The two main reasons are: the normal tissue volume overwhelms the effects of smaller volumes (tumor) and the vascular permeability in the healthy tissue is small. However, the vascular permeability in tumor has a high impact on the free VEGF concentration in the tumor as shown in figure 4B. For permeability higher than 4 × 10^-7 ^cm/s, the free VEGF level drops drastically in the tumor. This behavior is the result of the amount of VEGF molecules being cleared from the blood as they are transported from the tumor to the blood compartment. The plasma VEGF level is not highly affected by the increase of vascular permeability in the tumor because the clearance is directly proportional to the concentration and because the volume of the healthy tissue is bigger than that of the tumor. In any case, the plasma VEGF level does not exhibit a several-fold increase compared to the absence of tumor, even at high permeability (4 × 10^-5 ^cm/s which is an upper range of vascular permeability for VEGF in tumor found in the literature). This means that the vascular permeability in tumor increase alone cannot explain the several-fold increase of plasma VEGF level in cancer patients [11].

Over a range of clearance rates from 0.0146 and 0.0347 min^-1 ^[32], there is little variation in the free VEGF concentration (Figure 4C) and this also cannot explain the several-fold increase in plasma VEGF reported in cancer patients [11]. Increasing the NRP1 density in the tumor by an order of magnitude only decreases the tumor VEGF at high vascular permeability in tumor.

#### Increasing vascular permeability of healthy tissue increases blood and tumor VEGF levels

For high vascular permeability of healthy tissue (4 × 10^-6 ^cm/s – higher than that observed in vivo), the free VEGF concentration in plasma would be increased approximately four- to five-fold compared to baseline (Figure 4D). Therefore, the vascular permeability of healthy tissue may increase at sites distant from the tumor (possibly due to a feed-forward effect of VEGF-induced vascular permeability), leading in turn to a total increase of VEGF concentration in the plasma or the additive effects of increased VEGF secretion, vascular permeability in tumor, and tumor mass may explain the several-fold increase in plasma VEGF in cancer patients as reported in the literature [11].

#### Distribution of free vs. receptor- and HSPG-bound VEGF in healthy and diseased tissues

**Figure 5:**
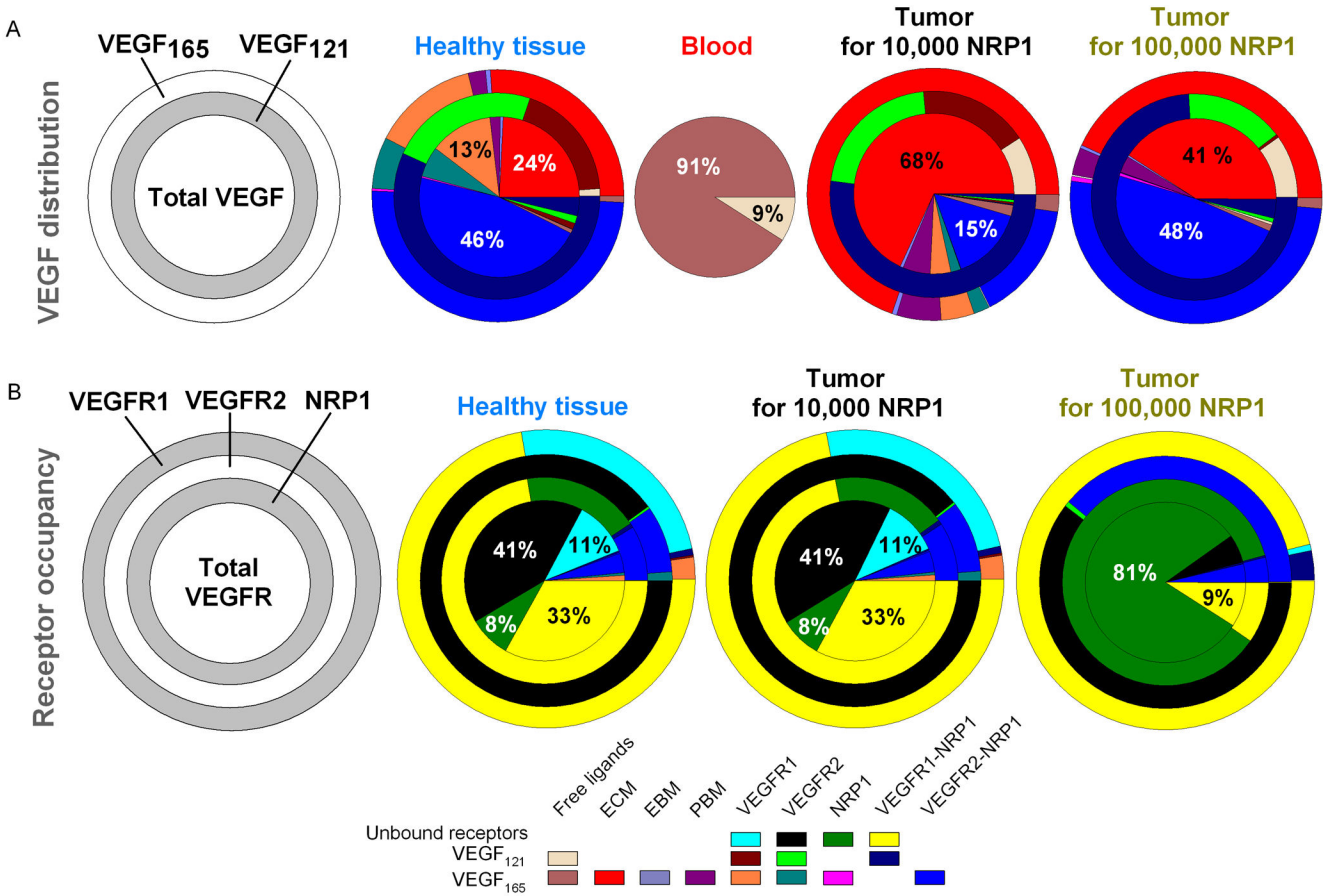
**Distribution of VEGF and its receptors for each tissue**. The diseased compartment represents a 4-cm diameter tumor. Vascular permeability of healthy tissue, $k_{p}^{N}$ = 4 × 10^-8 ^cm/s; vascular permeability of the tumor $k_{p}^{D}$ = 4 × 10^-7 ^cm/s; VEGF plasma clearance *c*_*V *_= 0.0206 min^-1 ^[28]; VEGFR1 = 10,000 and VEGFR2 = 10,000 molecules/endothelial cell; NRP1 = 10,000 molecules/endothelial cell in the healthy tissue; VEGF_165 _secretion rate in healthy tissue *q*_*N *_= 0.102 molecule/cell/s; tumor VEGF_165 _secretion rate *q*_*D *_= 0.076 or 0.025 molecule/cell/s for 10,000 (written in black) or 100,000 (written in dark yellow) NRP1 in tumor respectively. **A**, From center out, discs represent: total VEGF, VEGF_121 _and VEGF_165 _distributions. In the healthy tissue, about half of the total VEGF distribution is in the form of the ternary complex VEGF_165 _bound to VEGFR2 and NRP1, leaving 24% bound to the ECM. In the tumor, most total VEGF is bound to the ECM (68% and 41% for 10,000 and 100,000 NRP1 in tumor, respectively). Most of the remaining total VEGF population is in the form of the ternary complex VEGF_165 _bound to VEGFR2 and NRP1 (15% and 48% for 10,000 and 100,000 NRP1 in tumor, respectively). Most of VEGF_121 _isoform is bound to VEGFR1 and NRP1 simultaneously. The vast majority of the free VEGF distribution in the blood is in the isoform VEGF_165 _(91%). **B**, Receptor occupancy. From center out, discs represent: overall receptor, NRP1, VEGFR2, VEGFR1 occupancies. The initial receptor densities dictate the receptor occupancies. For identical NRP1 receptor densities (10,000), the healthy tissue and the tumor have the same receptor occupancies: 60% of all the receptors are in their free states and 33% are in the complex form VEGFR1-NRP1. If the NRP1 density is increased by 10-fold in the tumor, most of the total receptors are free NRP1 (81%) and a small fraction is bound to VEGFR1 (9%).

In the above sections, we presented results of computer simulations for free VEGF in the tissue and blood compartments. To understand the total balance of VEGF in the body, it is also important to assess the amounts of VEGF bound to the receptors on the endothelial cells and to the HSPG sites in the ECM and basement membranes. The VEGF distribution is shown in Figure 5A. For the parameters specified in the legend, 93% of total VEGF in the healthy tissue is VEGF_165_. The model revealed that up to half of the VEGF distribution in the healthy tissue and the tumor with 100,000 NRP1 per endothelial cell, is in the form of a complex where VEGF_165 _is bound to VEGFR2 and NRP1 simultaneously. In the tumor, 41 to 68% (depending on the NRP1 density) of the VEGF population, is VEGF_165 _bound to the ECM while it represented only a quarter in the healthy tissue. Finally, the vast majority of free VEGF in the blood is VEGF_165 _(91%), regardless of the density of NRP1 in the tumor.

#### The majority of cell surface receptors in healthy tissue and tumor receptors are not ligated

The receptor occupancy in the tumor with density of 10,000 NRP1 per endothelial cell is qualitatively similar to that in the healthy tissue (Figure 5B). In these tissues, most VEGFR1 and most NRP1 are present as the VEGFR1-NRP1 complex. Most VEGFR2 is unbound. The majority of ligated VEGFR2 is in the VEGF_165_-VEGFR2-NRP1 complex. A ten-fold increase in NRP1 density in the tumor causes uncomplexed NRP1 to dominate.

#### Concentration of free receptors and matrix components

**Figure 6:**
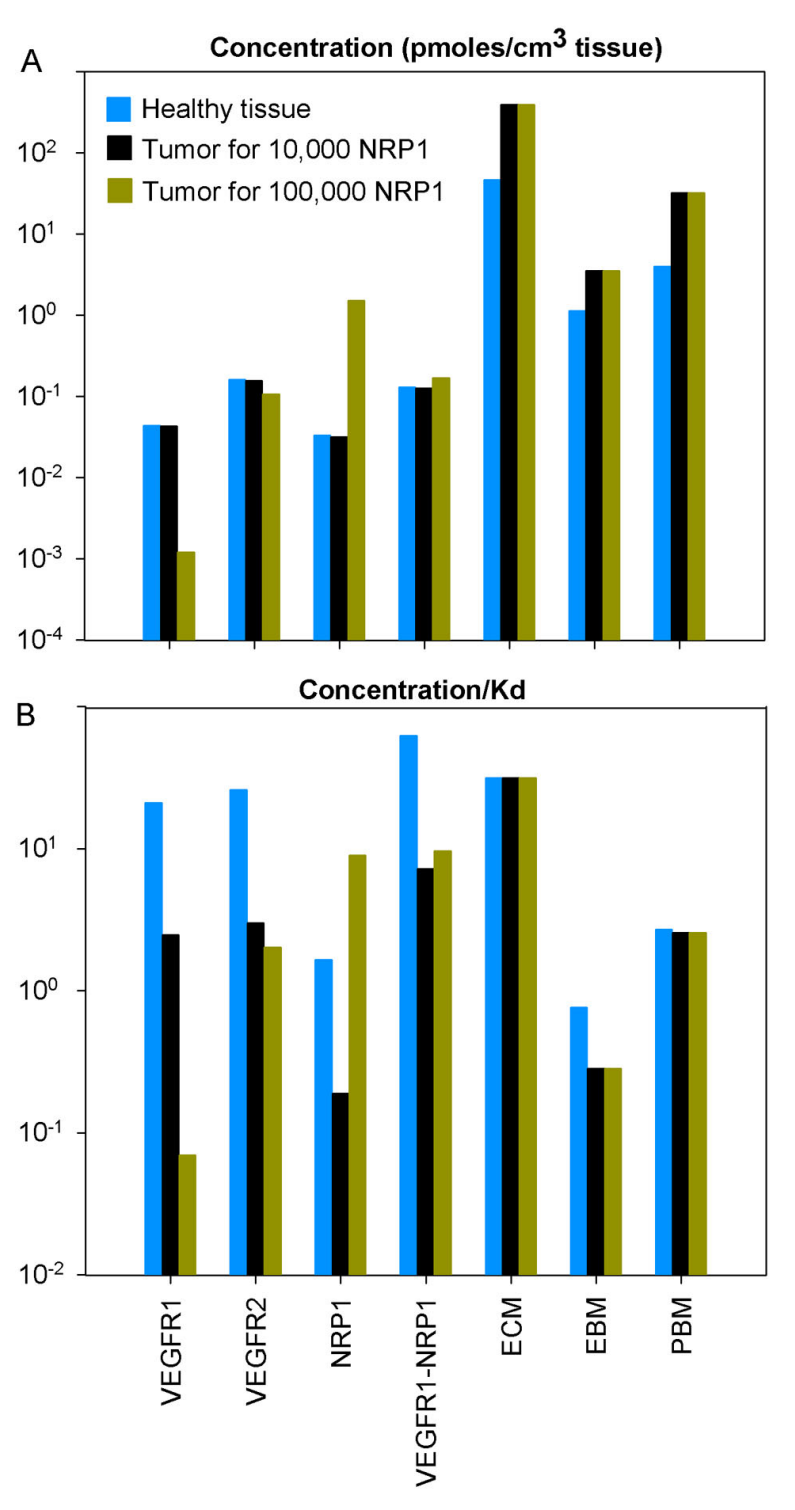
**Concentration of free receptors and matrix components and relative probabilities of VEGF binding**. The diseased compartment represents a 4-cm diameter tumor. Vascular permeability of the healthy tissue, $k_{p}^{N}$ = 4 × 10^-8 ^cm/s; vascular permeability in the tumor $k_{p}^{D}$ = 4 × 10^-7 ^cm/s; VEGF plasma clearance *c*_*V *_= 0.0206 min^-1 ^[28]; VEGFR1 = 10,000 and VEGFR2 = 10,000 molecules/endothelial cell; NRP1 = 10,000 molecules/endothelial cell in the healthy tissue; VEGF_165 _secretion rate in healthy tissue *q*_*N *_= 0.102 molecule/cell/s; tumor VEGF_165 _secretion rate *q*_*D *_= 0.076 or 0.025 molecule/cell/s for 10,000 (black bars) or 100,000 (dark yellow bars) NRP1 in tumor respectively. **A**, Concentration of free receptors and matrix components. For the same NRP1 density in both tissues, the healthy tissue and tumor have the same concentration of free receptors. The occupancy of the matrix components (ECM, PBM, EBM) is insensitive to the NRP1 density in the tumor. **B**, Relative probabilities of VEGF binding to receptors and matrix components (concentration/K_d_). The propensity to bind to receptors is much higher in healthy tissue than in tumor. The likelihood of binding to the matrix components, however, is very similar in the healthy tissue and tumor regardless of the NRP1 density.

Figure 6A compares the concentration of free receptors and free matrix components. In particular, we see that most of available binding sites are in the ECM, EBM and PBM. The available binding site concentrations of the matrix components are independent of the NRP1 density in the tumor.

#### Relative probabilities of VEGF binding to receptors and matrix components

The relative probabilities are expressed as concentration divided by K_d_. The propensity to bind to VEGFR1 and VEGFR2 is much higher in healthy tissue than in tumor (Figure 6B). In particular, the higher the NRP1 density in tumor, the less the binding to VEGFR1 and VEGFR2. However, the propensity to bind to NRP1 is much higher in tumor for a density of 100,000 NRP1 per endothelial cell. The probability to bind to the ECM is much higher than that of binding to the PBM or EBM because the volume of ECM is bigger.

#### Flows of VEGF in the body at steady state

**Figure 7:**
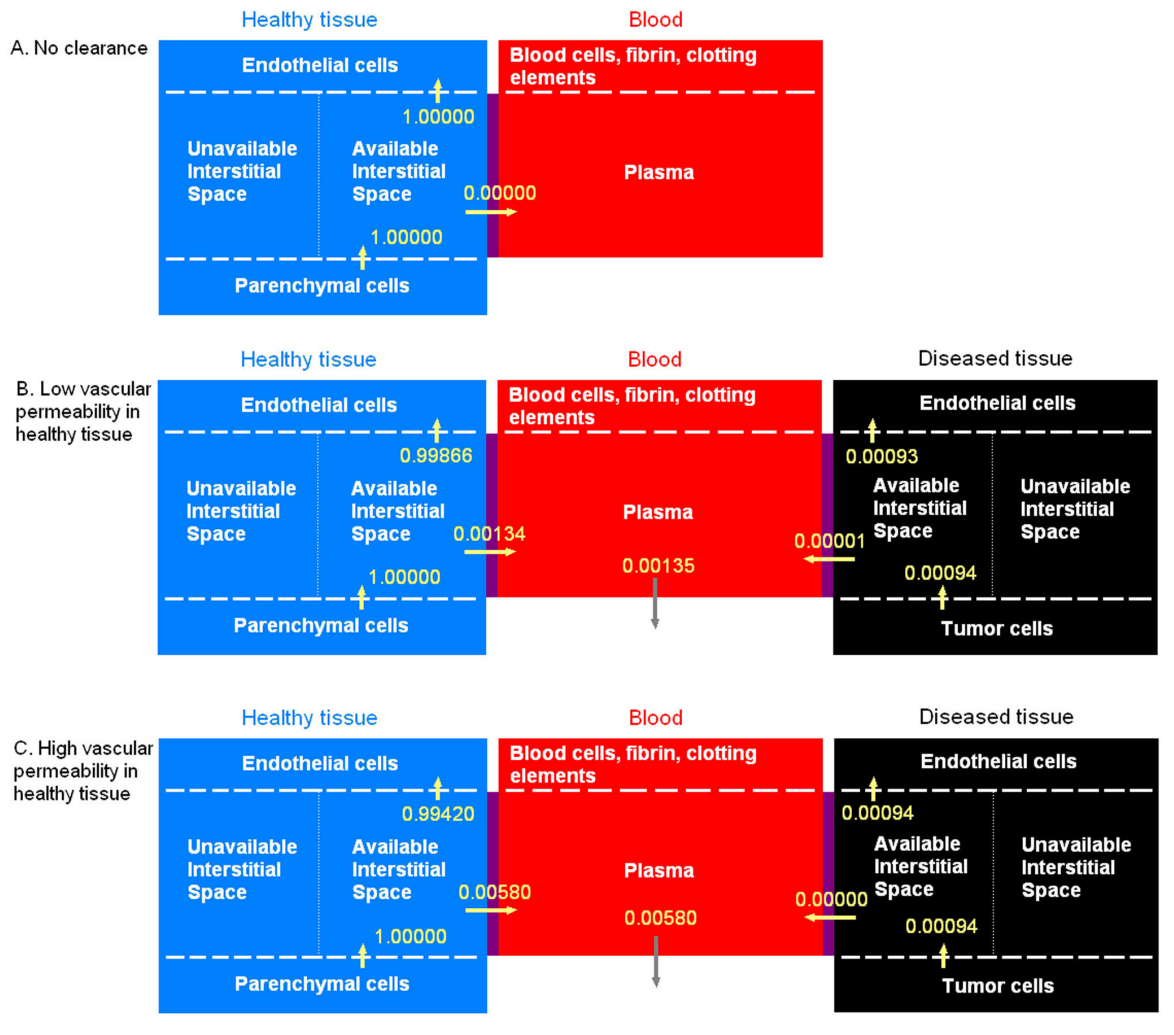
**Flows of VEGF in the body at steady state**. The flows are normalized to the moles of VEGF secreted per unit time in the normal tissue. Units before normalization: moles/s. VEGF_165 _secretion rate in healthy tissue *q*_*N *_= 0.102 molecule/cell/s; VEGFR1 = 10,000, VEGFR2 = 10,000 molecules/endothelial cell; NRP1 = 10,000 molecules/endothelial cell in the healthy tissue. **A**, In the absence of diseased tissue and clearance, the internalization of VEGF balances the secretion. There is no net flow between the two compartments. Vascular permeability of the healthy tissue, $k_{p}^{N}$ = 4 × 10^-8 ^cm/s; **B**, In the presence of a tumor and of VEGF clearance, most VEGF that has been secreted is internalized after binding to receptors (99.866%). A small fraction penetrates the bloodstream. Most of VEGF secreted in the tumor is being internalized after binding to receptors (99.150%). This configuration corresponds to low vascular permeability in healthy tissue in Figure 4D. The diseased compartment represents a 4-cm diameter tumor; vascular permeability of the healthy tissue, $k_{p}^{N}$ = 4 × 10^-8 ^cm/s; vascular permeability in the tumor $k_{p}^{D}$ = 4 × 10^-7 ^cm/s; VEGF_165 _secretion rate in tumor *q*_*D *_= 0.076 molecule/cell/s; VEGF plasma clearance *c*_*V *_= 0.0206 min^-1 ^[28]; NRP1 = 100,000 molecules/endothelial cell in the tumor. **C**, At higher vascular permeability in the healthy tissue $k_{p}^{N}$ = 4 × 10^-6 ^cm/s, the net flow of VEGF entering the bloodstream from the healthy tissue increases to 0.580%. The internalization of receptors in the healthy tissue decreases (99.420%) while the internalization of receptors in the tumor increases (99.861%). This configuration corresponds to high vascular permeability in healthy tissue in Figure 4D. The diseased compartment represents a 4-cm diameter tumor; vascular permeability $k_{p}^{D}$ = 4 × 10^-7 ^cm/s in the tumor and $k_{p}^{N}$ = 4 × 10^-8 ^cm/s in healthy tissue; VEGF_165 _secretion rate in tumor *q*_*D *_= 0.076 molecule/cell/s; VEGF plasma clearance *c*_*V *_= 0.0206 min^-1^; NRP1 = 100,000 molecules/endothelial cell in the tumor.

Figure 7 shows the flows (moles of VEGF per unit time) in the compartments normalized to the number of moles of VEGF secreted per unit time in the healthy tissue. We first consider a healthy subject (no diseased tissue compartment). In the absence of clearance, at steady state, there is as much VEGF secreted as internalized (Figure 7A). There is no net flow between the normal tissue and the blood. This configuration corresponds to Figure 2A in the absence of clearance (purple line). Figure 7B shows the representation of a cancer patient. At low vascular permeability in the healthy tissue, almost 99.9% secreted VEGF is internalized at steady state. Because a small fraction of VEGF from the healthy tissue enters the bloodstream (less than 0.2%), a small fraction is cleared from the plasma. If the vascular permeability in the healthy tissue is increased by an order of magnitude (Figure 7C), a larger percentage of VEGF enters the bloodstream (0.58%) which leads to a larger percentage of VEGF cleared from the plasma. Because the percentage of VEGF extravasating into the healthy tissue is increased, the internalization of VEGF in the healthy tissue is only slightly affected by the increase in vascular permeability in the healthy tissue. Most of VEGF secreted in the tumor is internalized at steady state. However, when increasing the vascular permeability in healthy tissue, some VEGF extravasates into the tumor canceling out the percentage of VEGF that has entered the bloodstream from the tumor. Essentially, since the net flow is zero in this configuration, the diseased compartment does not play any significant role in the VEGF in the blood or in the healthy tissue. This explains why Figure 4D is similar to Figure 2B.

## Discussion

The compartment model described here is a useful tool to simulate physiological and pathological situations involving VEGF. It provides informative quantitative biological details such as VEGF distributions in tissue and in blood that are currently not accessible by direct experiments, as well as the sensitivity of VEGF distribution to specific biological parameters.

Even though, at this stage, the model considers two isoforms of the VEGF-A family, the model can be readily extended to introduce additional factors, such as the isoform VEGF_189 _that could also play an important role in angiogenesis. For example, it was shown that the VEGF_189 _represents the highest increase in protein levels amongst the VEGF-A isoforms in the course of exercise training (3-fold for VEGF_189 _compared to 2.2-fold for VEGF_165 _and VEGF_121_) [34]. Receptors and co-receptors could also be added: soluble VEGFR1 (sFlt-1) or Neuropilin-2 (NRP2), for example, could also play significant roles in angiogenesis. Molecules that do not play a role in angiogenesis but compete for the receptor binding and therefore alter the VEGF distribution could also be taken into account. For instance, the virus-encoded VEGF-E proteins bind with high affinity to VEGFR2 but do not bind to VEGFR1, and VEGF-B and PlGF compete with VEGF-A for binding to VEGFR1 [3].

The model takes into account VEGF receptors on the abluminal surface of the endothelial cells. There is some evidence that these receptors may be present on their luminal surfaces as well [17]; however, to our knowledge, no quantification is available. To take these receptors into account would require the introduction of receptors in the blood compartment and could potentially change the dynamics of the system. Since the VEGF secretion rates were fine-tuned based on the steady-state concentration of VEGF in the plasma found in the literature [11], the addition of the VEGF receptors on the luminal surface of the endothelial cells may therefore require re-adjustment of the secretion rates values.

For simplicity, healthy tissue is represented as approximately 65 kg of tissue whose parameters are representative of human vastus lateralis skeletal muscle, since this is one of the few tissues characterized. It could be of interest to add the kidneys or liver and explicitly illustrate clearance from these organs. More generally, clearance from healthy tissue could be added. The addition of bones and poorly-vascularized organs could also play an important role since secretion of VEGF apparently does not occur in these tissues. The bone marrow could also be an important component to add to the model as it is the site where pro- and anti-angiogenic factors are segregated into separate platelet *α*-granules to be transported within the bloodstream and possibly released at the site of the tumor [35] thus protecting VEGF from binding to receptors on the luminal side of the endothelial cells or binding to anti-VEGF agents present in the bloodstream or extravasating. This crucial point leads to the introduction of the platelets as well. Not only are platelets a vector for VEGF transport in the bloodstream, they have also been shown to be a location where the binding with VEGF monoclonal antibodies, used in anti-angiogenic therapies, takes place [36].

Due to lack of experimental data in vivo, we consider *k*_*int *_to be the same in tumor and normal tissues. Experimental evaluation of the internalization rates of the receptors in vivo in healthy tissue and tumor would improve the accuracy of the model.

In our current model, vascular permeability is independent of VEGF concentration. However, VEGF increases permeability in pathological angiogenesis where the blood vessels become leaky. Therefore, the model should define the permeability as a function of the VEGF concentration. Another factor that could be significant is the transport of VEGF via the lymphatics.

## Conclusion

A compartmental model was formulated to represent both VEGF transport throughout the entire human body and the distribution of free and bound VEGF at the molecular level in tissues. Blood and tissue are interconnected by vascular permeability for VEGF transport.

In the healthy subject, in the absence of clearance, free VEGF in the plasma follows that in the available interstitial fluid volume in the healthy tissue. When clearance is introduced, free VEGF levels in tissue and blood are still approximately proportional to the VEGF secretion rate. However, free VEGF concentration is lower in plasma than in tissue. We also demonstrated that the internalization of the receptors decreases as the permeability increases, maintaining the free VEGF level constant in the normal tissue while the concentration in the blood increases as well.

The model was used to determine variations of VEGF levels during an exercise training experiment. We simulated a 3-hour two-legged knee extension, studied by Jensen *et al*. [13]. After a 6-hr upregulation of VEGF secretion, the VEGF concentration returns to baseline after another 6 hours, assuming proportionality between mRNA and VEGF protein level. The model predicted a time lag in VEGF levels between tissue and blood during the transition periods. This has implications for measurements as blood samples could exhibit higher VEGF levels than tissue samples.

In pathological cases, a third compartment representing diseased tissue was added. In our simulations, this diseased tissue was chosen to be a 4-cm diameter tumor located in the breast. We investigated the possible causes of the several-fold increase in plasma VEGF in cancer patients reported in the literature. Free VEGF concentration in healthy tissue remained constant during all the performed simulations because the volume of that tissue was much larger than that of the tumor and the vascular permeability of healthy tissue is low. The tumor compartment was generally the most sensitive to the change of VEGF secretion rates in the tumor and the vascular permeability in the tumor. These variations did not affect the plasma VEGF levels significantly. However, increasing vascular permeability in healthy tissue increased plasma VEGF levels, suggesting that, apart from additive effects (such as an increase in VEGF secretion rate, combined with higher vascular permeability in the tumor and increase tumor volume), a change in vascular permeability in the healthy tissue could explain the plasma VEGF increase in cancer patients.

The model revealed that about half of the VEGF distribution is in the form of a ternary complex where VEGF_165 _is bound to VEGFR2 and NRP1. In the tumor, most of the other half of the VEGF population was VEGF_165 _bound to the ECM while it represented only a quarter in the normal tissue. This led to a low amount of VEGF_165 _bound to VEGFR1 and VEGFR2 in the tumor whereas it represents about 20% in the normal tissue. Most of VEGFR2 is in its free state while most VEGFR1 and NRP1 are present as the VEGFR1-NRP1 complex. The available binding site concentrations of the matrix components are independent of the NRP1 density in the tumor. Finally, the model showed that the higher the NRP1 density in tumor, the less the binding to VEGFR1 and VEGFR2.

This model has provided new insights on molecular distribution and biological details that cannot be easily assessed experimentally. The adaptability of the compartmental model allows the simulation of human or animal subjects and VEGF-dependent diseases as long as the biological properties of the studied tissue are available. This model can be extended by including new molecular species, taking into account platelets and leukocytes as VEGF carriers, or biophysical processes that could intervene in VEGF transport or VEGF binding. In particular, the model presented here could serve as a basis for devising pro- and anti-angiogenic therapies and testing their potential effects on the VEGF distribution in the human body.

## Glossary

The units are given for the healthy tissue and tumor compartments unless specified.

[species] Concentration of species (in mol/cm^3 ^tissue)

*q*_*V*_ VEGF secretion rate (in mol/cm^3 ^tissue/s)

$k_{pV}^{IJ}$ Permeation rate of VEGF from compartment *I *to compartment *J *(in cm/s)

*S*_*R *_Insertion rate of the receptors R (in mol/cm^3 ^tissue/s)

*k*_*on *_Association rate constant (in (mol/cm^3 ^tissue)^-1^/s)

*k*_*off *_Dissociation rate constant (in s^-1^)

*k*_*c *_Rate constant of cell surface receptor coupling (in (mol/cm^3 ^tissue)^-1^/s)

*k*_*dissoc *_Dissociation rate constant of coupled receptors (in s^-1^)

*k*_int _Internalization rate constant for cell surface receptors (in s^-1^)

*c*_*V *_Clearance rate of VEGF from the plasma (in s^-1^)

*S*_*IJ *_Surface between compartment I and compartment J (in cm^2^)

*U*_*I *_Volume of compartment I (in cm^3^)

## Authors' contributions

MOS carried out the calculations, performed the simulations and wrote the first version of the manuscript. FTHW, FMG and ASP participated in the design of the study and the model formulation, analysis of the results and writing and editing the manuscript. All authors read and approved the final manuscript.
